# Integrated Multimodal Imaging of Dynamic Bone-Tumor Alterations Associated with Metastatic Prostate Cancer

**DOI:** 10.1371/journal.pone.0123877

**Published:** 2015-04-10

**Authors:** Jean-Christophe Brisset, Benjamin A. Hoff, Thomas L. Chenevert, Jon A. Jacobson, Jennifer L. Boes, Stefanie Galbán, Alnawaz Rehemtulla, Timothy D. Johnson, Kenneth J. Pienta, Craig J. Galbán, Charles R. Meyer, Timothy Schakel, Klaas Nicolay, Ajjai S. Alva, Maha Hussain, Brian D. Ross

**Affiliations:** 1 Department of Radiology, University of Michigan, Ann Arbor, Michigan, United States of America; 2 Department of Radiation Oncology, University of Michigan, Ann Arbor, Michigan, United States of America; 3 Department of Biostatistics, University of Michigan, Ann Arbor, Michigan, United States of America; 4 Division of Hematology/Oncology, University of Michigan, Ann Arbor, Michigan, United States of America; 5 Department of Radiotherapy, University Medical Center Utrecht, Utrecht, The Netherlands; 6 Department of Biomedical Engineering, Eindhoven University of Technology, Eindhoven, The Netherlands; University of Kentucky College of Medicine, UNITED STATES

## Abstract

Bone metastasis occurs for men with advanced prostate cancer which promotes osseous growth and destruction driven by alterations in osteoblast and osteoclast homeostasis. Patients can experience pain, spontaneous fractures and morbidity eroding overall quality of life. The complex and dynamic cellular interactions within the bone microenvironment limit current treatment options thus prostate to bone metastases remains incurable. This study uses voxel-based analysis of diffusion-weighted MRI and CT scans to simultaneously evaluate temporal changes in normal bone homeostasis along with prostate bone metatastsis to deliver an improved understanding of the spatiotemporal local microenvironment. Dynamic tumor-stromal interactions were assessed during treatment in mouse models along with a pilot prospective clinical trial with metastatic hormone sensitive and castration resistant prostate cancer patients with bone metastases. Longitudinal changes in tumor and bone imaging metrics during delivery of therapy were quantified. Studies revealed that voxel-based parametric response maps (PRM) of DW-MRI and CT scans could be used to quantify and spatially visualize dynamic changes during prostate tumor growth and in response to treatment thereby distinguishing patients with stable disease from those with progressive disease (p<0.05). These studies suggest that PRM imaging biomarkers are useful for detection of the impact of prostate tumor-stromal responses to therapies thus demonstrating the potential of multi-modal PRM image-based biomarkers as a novel means for assessing dynamic alterations associated with metastatic prostate cancer. These results establish an integrated and clinically translatable approach which can be readily implemented for improving the clinical management of patients with metastatic bone disease.

**Trial Registration:**

ClinicalTrials.gov NCT02064283

## Introduction

Bone metastasis is the hallmark of prostate cancer and is a major cause of morbidity and mortality [1,2]. It is found in over 90% of men with castration-resistant disease [3,4] and in most patients postmortem [5,6] Clinical response criteria used for assessment of treatment efficacy are based upon changes in the anatomical size of the tumor. Recent changes in these criteria have been detailed as part of the updated Response Evaluation Criteria in Solid Tumors (RECIST 1.1) which consider lytic or mixed lytic-blastic bone metastases with soft tissue masses greater than 1 cm to be measurable disease, but blastic bone lesions are still considered non-measurable [7]. The use of imaging in the clinical management of bone metastasis has traditionally relied predominantely on bone scintigraphy using ^99m^Tc-methyl diphosphonate [8–10]. Plain film radiographs, MRI and more recently PET [11,12] have been used adjunctly. While assessment in the response of primary or metastatic cancers within the skeletal system has been a longstanding problem, alternative strategies including functional and molecular imaging approaches are being pursued [12–14]. However, traditional imaging relies upon either visual intrepretation of acquired scans by a musculoskeletal radiologist or by whole volume quantification of mean values of voxels contained within a region of interest (i.e. a tumor). Furthermore, integration of the information available from multimodal images on a voxel-by-voxel basis to assess the spatiotemporal effects of tumor growth and response to therapy has not been attempted to date.

Diffusion-weighted MRI (DW-MRI) has been reported as a tool for assessing cancer response to therapy as it is able to quantify the random (i.e., Brownian) motion of water molecules within tissue [15–18]. Water diffusion values are reduced in the presence of cellular membranes which impede the motion of water molecules. Effective treatments result in a loss in the number of tumor cells thus reducing restrictive barriers and allowing for more rapid water mobility (i.e., diffusion). DW-MRI is able to capture these subtle changes by quantifying water mobility as the apparent diffusion coefficient (ADC) in tumors. The application of DW-MRI for tumor treatment response assessment was initially described using a 9L glioma model [19] and was successfully extended in preclinical studies evaluating the response to a variety of anticancer interventions [20–23]. Further evolution in image post-processing of tumor ADC values was undertaken for assessing treatment response through the development of a voxel-by-voxel algorithm to account for intratumor heterogeneity, an approach termed the functional diffusion map (fDM) [24–27]. The fDM approach tracked changes in the ADC values of individual tumor voxels over time in patients with primary malignant brain tumors as well as a brain tumor model where the amount of fDM-detected change in diffusion values was shown to correlate with overall survival [27–34].

More recently, successful use of DW-MRI and the fDM metric for providing early indication of treatment response in preclinical models as well as patients diagnosed with metastatic prostate cancer to the bone have been reported [27,35–37]. Furthermore, extension of the voxel-based image analysis approach was significantly advanced by showing that it could be generally applied to a variety of imaging modalities including perfusion MR, PET and CT and was re-termed the parametric response map (PRM) [38–42]. In particular, application of PRM analysis to CT images obtained from a rat model of osteoporosis revealed that this approach could provide the ability to spatially track and quantify changes in bone mineral density over time [43].

In this present study we investigated the use of multimodal PRM imaging biomarkers for their ability to quantify dynamically changing tumor-host interactions by monitoring tumor treatment response (PRM_ADC_) along with changes in bone density (PRM_CT_) using *in vivo* mouse models and a pilot clinical trial involving patients with prostate cancer and bone metastasis. The mouse studies provided an opportunity to evaluate the impact of osteolytic and osteoblastic tumor models on the imaging metrics along with the effect of therapeutic intervention and results revealed that the multimodal approach could detect spatiotemporal tumor and bone alterations. Furthermore, results from the clinical study provide initial results showing that this approach can be successfully translated into the clinical setting allowing for multimodal disease monitoring of dynamic bone microenvironmental alterations associated with metastatic prostate cancer to the bone. Integration of the PRM imaging results into future clinical workflow will impact our ability to assess putative therapies in clinical trials as well as in routine patient management.

## Materials and Methods

The protocol for this trial and supporting CONSORT checklist are available as supporting information; see S1 CONSORT Checklist and S1 Protocol.

### Study 1: PC3 Tumor Response to Docetaxel and Radiation

The therapeutic effects of docetaxel and ionizing radiation (IR) were evaluated in an intra-tibial model of prostate cancer metastasis to the bone using DW-MRI. Androgen independent human prostate cancer [44] cells were purchased from American Type Culture Collection and grown in RPMI 1640 with 10% fetal bovine serum.

All studies involving the use of mouse protocols were approved by The University of Michigan Committee on Use and Care of Animals. Male severe combined immunodeficient (SCID) mice were included in the study at 4–6 weeks of age. Mice were anesthetized by intraperitoneal injection of a mixture of ketamine (100 mg/kg) and xylazine (10 mg/kg). A Hamilton syringe with a 28-gauge needle was inserted in the middle of the patella ligament through the tibial crest epiphysis and growth plate. To prevent pain, Carprofen (5mg/kg) was injected subcutaneously. PC3 cells (5x10^5^) suspended in 10µl of media were injected into the trabecular bone of the tibial metaphysis. Sham surgeries were performed on the left leg using an identical procedure by injecting media only.

When tumor volumes reached 7 to 15 mm^3^, as determined by MRI, animals were entered into one of four treatment groups: docetaxel (n = 6), IR (n = 6), docetaxel + IR (n = 6), or vehicle control (n = 8). Docetaxel treatment at a dose of 20 mg/kg in 10% DMSO was administered weekly via intraperitoneal injection for three weeks. The effect of IR on PC3 tumors was evaluated alone and in combination with docetaxel at a dose of 2 Gy/day 5 times per week for two weeks using an IC-320 Specimen Irradiation System. For combination therapy, animals were first treated with docetaxel followed by IR 4 hours later.

MRI scans were acquired on days 0, 1, 4, 7, 11, 14, and then weekly until tumors surpassed a pre-defined terminal volume increase of 400% from initial volume. Imaging was performed using a 9.4T Agilent system. DW-MR was accomplished using a spin-echo sequence, with a navigator echo and gradient waveforms sensitive to isotropic diffusion [45] as previously described.

### Study 2: Early Bone Protection with Bisphosphonates

Bisphosphonate treatment using zoledronic acid early after tumor model induction was evaluated in a mouse model using x-ray micro-computed tomography (µCT) of the bone to assess the sensitivity of PRM_CT_ for the detection of bone remodeling. PC3 prostate cancer cells were cultured and implanted in mice as described above in Study 1.

The bisphosphonate zoledronic acid was evaluated as a bone-protective agent, administered early after initiation of the bone tumor (n = 8) and compared to vehicle controls treated with PBS (n = 5). Treatments of 5mg/kg began 3 days post-implantation (day 0 for the study) and were administered twice weekly for four doses (days 0, 3, 7, and 11).

Acquisition of µCT scans were twice weekly until study end. Images were acquired using a Siemens Inveon system (56-µm voxel size). DW-MR acquisitions were as in Study 1.

### Study 3: Chemotherapy of Osteoblastic Lesions

Osteoblastic lesions derived from LAPC-9 cell implantation [46,47]were treated with docetaxel (n = 3) or vehicle (n = 3) as in Study 1 to compare signatures of osteolytic/-blastic tumor growth and response using MR and CT-derived imaging biomarkers. Cells were maintained by propagation as subcutaneous xenografts in SCID mice until needed for implantation [48]. Immediately prior to cell implantation, LAPC-9 tissue was harvested and treated with collagenase to facilitate cell implantation into male SCID mice. Docetaxel was administered weekly at a dose of 20mg/kg via intraperitoneal injection.

DW-MR scans were acquired weekly until the end of the study as described in Study 1. Changes in tumor volume and ADC were monitored to evaluate tumor treatment response. Bone response was evaluated using weekly µCT imaging to detect changes in mineral density in the vicinity of the tumor.

### Clinical Trial

Eligible patients required a confirmed diagnosis of prostate cancer, evidence of bone metastatic disease by bone scan for which initiation of systemic therapy would be undertaken. All patients underwent standard disease imaging with bone scan and CT of abdomen and pelvis. Patients with newly diagnosed metastatic disease underwent androgen deprivation while patients with castration resistant prostate cancer (CRPC) were treated with systemic non-hormonal therapy. The study was approved by the University of Michigan Institutional Review Board and all patients signed as IRB approved consent.

Image-derived metrics included volume percent change of the number of tumor voxels from registered diffusion MRI maps and CT images which were compared to clinical response outcomes to investigate the utility of PRM as an early predictor of therapy in these patients. Patients were separated into response groups based on the response criteria used in this trial where outcomes were defined by the following post-therapy PSA changes:

**Complete Response (CR):** Undetectable PSA (<0.2 ng/ml) confirmed by another PSA level at no less than 4 weeks.
**Partial Response (PR):** Decrease in PSA value by ≥ 50% confirmed by another PSA level at no less than 4 weeks.
**Stable Disease (SD):** Patients who do not meet the criteria for CR, PR or PD were considered stable.
**Progression (PD):** A 25% increase over baseline or measured nadir PSA level whichever is lower and an increase in the absolute value of PSA level by 5 ng/ml that is confirmed by another PSA level at no less than 4 weeks.

**Fig 1:**
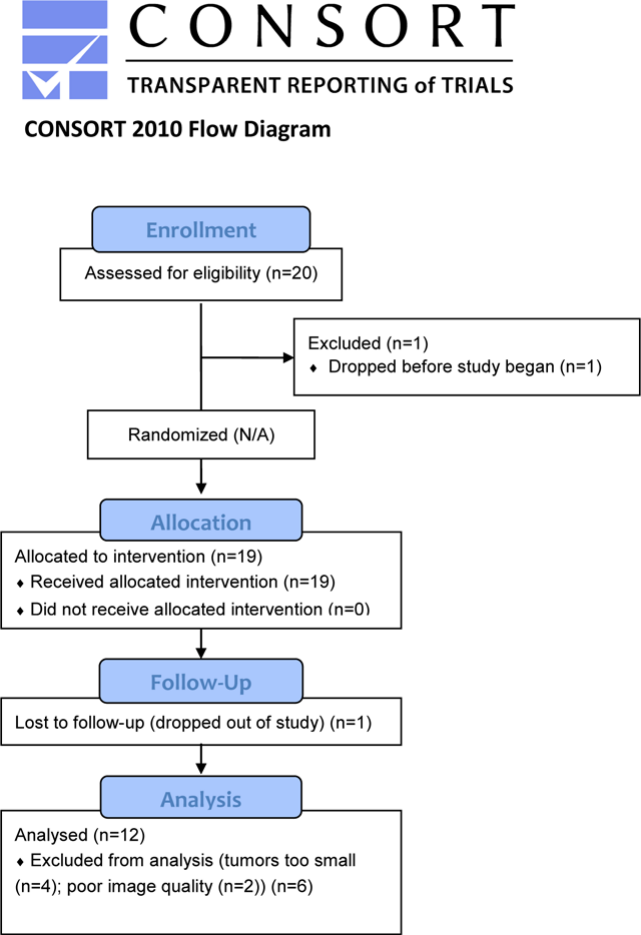
A CONsolidated Standards of Reporting Trials (CONSORT) flow diagram of the overall study patient population recruitment and disposition over the course of study.

Shown in Fig 1 is a CONsolidated Standards of Reporting Trials (CONSORT) flow diagram of the overall study patient population recruitment and disposition over the course of study. Of the twenty patients recruited and enrolled in this study, fifteen patients had CRPC and 5 patients had hormone sensitive disease. Eight of the 20 patients were not included in the analysis due to the following reasons: patient dropped out of study before treatment began (n = 1), poor image quality (n = 2), tumors were too small (n = 4), lack of follow-up scans (n = 1) thus a total of 12 patients were available for image analysis from this pilot study. Of the 12 patients 10 received chemotherapy and 2 patients were treated with hormone deprivation.

Baseline MRI was performed before therapy (week 0) and again at approximately 2 and 10 weeks post-treatment initiation using a clinical Philips 3T scanner. The MRI protocol included: 3-plane anatomical survey; Proton-Density; STIR Fat-Suppressed; T1-weighted, and DW scans.

Computed tomography (CT) scans consisted of pre-treatment acquisition and one additional scan collected as part of routine clinical care at approximately 10 week post-treatment initiation. Standard bone imaging protocols were used consistently for all patients, including helical acquisition and intravenous contrast.

Bone scans were obtained pre-treatment and again at approximately 10 week post-treatment initiation as part of routing clinical care. The ^99m^Tc-MDP bone scans were acquired following injection of 20–25 mCi of ^99m^Tc-MDP intravenously with imaging acquisition occurring following a 3–5 h uptake period.

### Quantitative Image Analysis

#### Tumor Volumes

Tumor volumes of interest (VOI) were determined by contouring the tumor on each slice of the high-b DW-MR image and integrated across slices to provide a volume estimate. Only tumors of 4 cm^3^ or greater were included in the clinical study to maintain a sufficient number of voxels and minimize partial-volume effects.

#### Apparent Diffusion Coefficient (ADC)

MR data was analyzed using software developed in MATLAB. ADC maps were generated using a two-point subsampling [49] of the signal decay curve using the following equation:

$$ADC_{1-2}\;=\;\frac{1}{\left(b_{2}-b_{1}\right)}\times \ln(\frac{s_{1}}{s_{2}})$$

where S_1_ and S_2_ are the signal intensities at b-values b_1_ and b_2_, respectively, and ADC_1–2_ is the apparent diffusion coefficient obtained using b_1_ = 120 and b_2_ = 1200 s/mm^2^ which desensitizes the diffusion measurement to local perfusion effects.

#### CT Hounsfield Units (HU)

CT images were calibrated to the Hounsfield scale using a water or water-equivalent phantom (= 0 HU) and surrounding air (= -1000 HU) on a linear scale. For mouse images, VOIs were generated to encompass the tibia from the tibia-fibula junction to the tibial plateau. VOIs for clinical images were contoured to encompass the bone surrounding the known location of the osseous lesion.

### Parametric Response Maps (PRM)

The Parametric Response Map is capable of detecting changes in quantitative imaging metrics on a voxel scale [24,25,39]. Image co-registration was performed using an automated iterative image transformation algorithm using the objective function of mutual information [50]. Each lesion was co-registered individually using either rigid-body transformation (CT) or a thin-plate spline warping interpolant (MRI).

After registration, each image sub-volume (voxel) is associated with two quantitative indices, one pre-treatment and the other post-treatment. Image voxels were statistically classified by their change over time (ΔX) using a pre-determined threshold (i.e., applying a 95% confidence interval of no change) into one of the following categories: increased (PRM_X+_, red), decreased (PRM_X-_, blue), or unchanged (PRM_X0_, green), where X denotes the quantitative index under analysis (i.e., ADC or HU). PRM analysis was applied to both ADC values as a measure of change in tumor cellularity [27,35–37] and HU values as a measure of bone mineral density in the vicinity of the tumor [43]. The threshold used for voxel classification was determined by the 95% confidence interval found in images obtained the same day (test-retest data), and varied by imaging modality and clinical/preclinical as follows: for ADC a threshold of 32 or 55 mm^2^/sec was determined and for HU a threshold of 391 or 100 HU was determined to be the upper and lower limits of the respective confidence limits for mice and human patients [27,35–37].

### Statistical Analysis

For statistical analysis, a normal distribution was assumed and comparisons between groups were performed at each time point using a two-tailed Student’s t-test (Microsoft Excel). Significant difference between groups was determined by p < 0.05 (denoted by * in all figures). All data was presented as mean±SEM.

## Results

### PRM_ADC_ Assessment of Metastatic Prostate Cancer to the Bone Treatment Response

**Fig 2:**
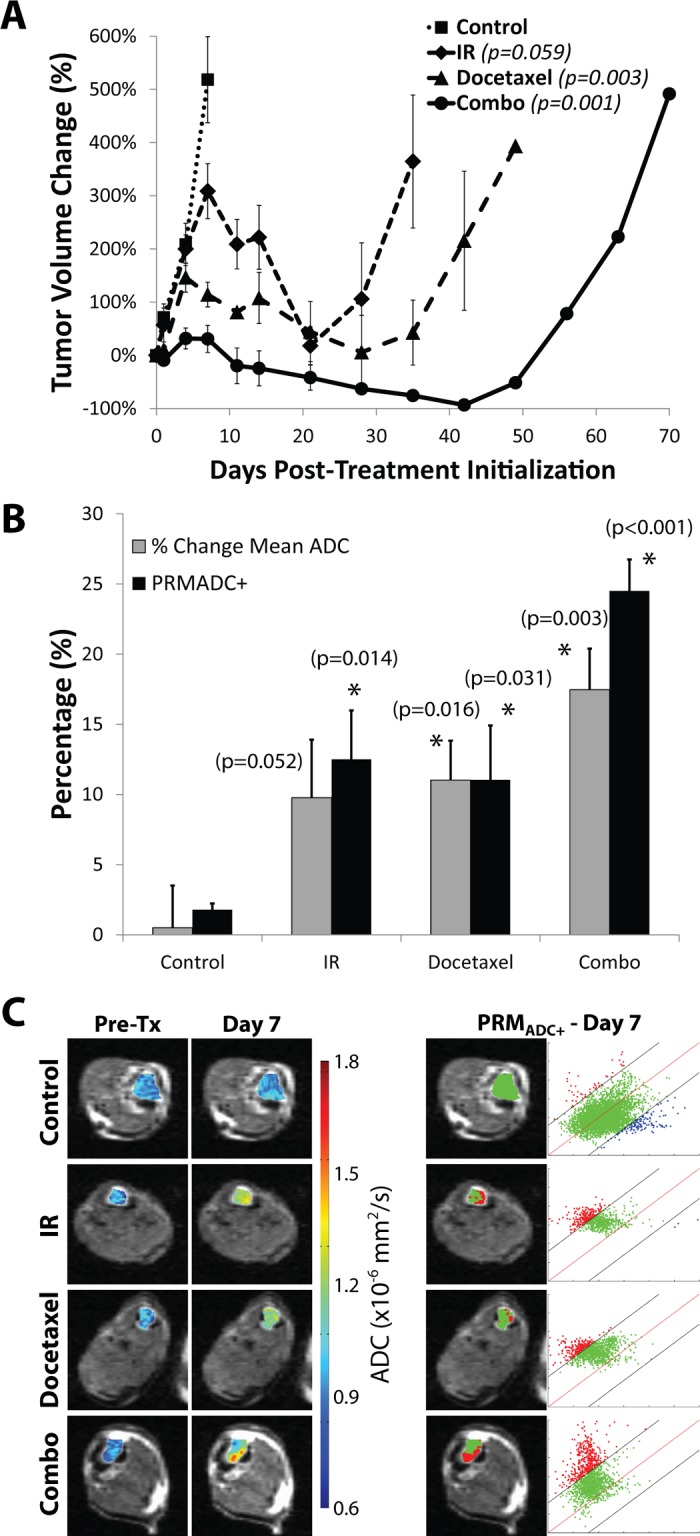
Intratibial PC3 tumor response to docetaxel (n = 6), radiation (IR, n = 6), or combination treatment (n = 6) shows an additive effect by anatomical and diffusion MRI compared to controls (n = 8). (A) Tumor volumes plotted over time (p-values in legend show significance versus controls at day 7) show greater cell kill in the combination group (circles, solid line) than either docetaxel (triangles, long-dashed line) or IR (diamonds, short-dashed line) treatment alone, and all treatments resulted in significant cell kill over controls (squares, dotted line). (B) Comparison of mean ADC change to PRM_ADC+_ at day 7 post-treatment-initialization resulted in no significant difference between measurements, but slightly elevated PRM_ADC+_ over mean ADC in the combination treatment (significant difference from controls: * (p<0.05)). (C) ADC color over overlays are shown in the left two columns for pre-treatment and day 7, and corresponding PRM_ADC_ overlay and scatterplot are shown on the right.

Trabecular bone tumors underwent treatment initiation within 4 weeks post-cell implantation. Tumor volumes of control animals showed continual growth throughout the study until an endpoint of +400% (i.e., 5 times the initial volume) was reached (Fig 2A). Animals treated with IR and docetaxel exhibited significant growth delays of about 30 and 40 days, respectively. Further enhancement of treatment efficacy was found in the combination therapy group with an extended growth delay of 60 days (Fig 2A).

DW-MRI was also evaluated for its ability to detect early response by evaluating the changes in tumor water diffusion values during the first week of therapy. As shown in Fig 2B and 2C, tumors were analyzed using both mean tumor ADC as well as the PRM voxel-based approach. Analysis of tumor ADC changes using PRM revealed a significant shift in the distribution of tumor voxels to higher ADC values (red voxels) moving above the 95% cutoff threshold which served as the biomarker metric of response. The PRM color overlay revealed heterogeneous changes in ADC values at 7 days post-treatment initiation within an individual tumor. PRM scatter plots (2C) were generated by plotting ADC values from each voxel on day 7 (y-axis) against day 0. The red line represents equality between measurements whereas the black lines represent the 95% confidence interval cutoff which was determined to be ±0.32x10^-3^ mm^2^/s. Control tumors exhibited little change in ADC over the first 7-day period post-treatment, as evidenced in the representative overlay (Fig 2C) by majority of green voxels. Conversely, representative overlays showing docetaxel and IR-treated animals demonstrate the substantial fraction of voxels that shifted to higher values at day 7 due to an increase in tumor ADC values. Animals treated with a combination of docetaxel and IR presented an even greater change in ADC than either treatment alone.

Changes in mean tumor ADC for each group showed similar magnitudes of response compared to PRM_ADC_ (Fig 2B and 2C). The mono-therapy groups were both found to have an approximate 10–12% increase in mean tumor ADC values at 7 days post-treatment initiation whereas the combined treatment group exhibited a 25% increase. The percent change in mean ADC as well as the PRM_ADC+_ values of the docetaxel, IR and combined treatment groups were all statistically different from the control group at day 7 (p<0.01).

### PRM_CT_ Assessment of Protective Bone Treatment from Metastatic Prostate Cancer

**Fig 3:**
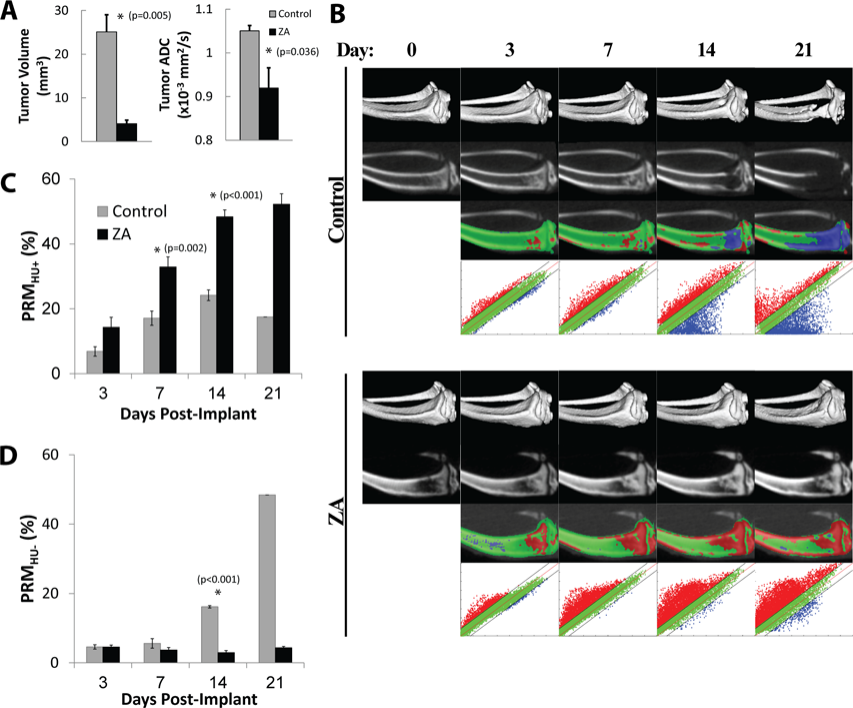
PC3 implantations treated with zoledronic acid (ZA, n = 4) show a bone-protective effect compared to controls (n = 8). (A) MRI tumor volume and ADC determined at day 21 post-treatment-initiation shows a retardation of tumor growth and significantly lower ADC in the zoledronic acid treated animals. (B) PRM_HU+_ bar plot shows significantly higher volume of bone that increased in density after treatment compared to controls. (C) PRM_HU-_ bar plot shows minimal loss of bone in the ZA-treated group, compared to progressively increasing bone loss in the controls. (D) Representative images for a control (top) and ZA-treated (bottom) mouse showing (from top to bottom) an isosurface, CT slice, PRM overlay, and PRM scatterplot from pre-treatment to 21 days post-treatment.

PRM was applied to CT images to monitor bone tissue density changes during early protective treatment of PC3 tumors with ZA and compared to untreated animals soon after implantation of tumor cells. ZA was used as a treatment to evaluate the ability of PRM_CT_ to detect the effects of a bisphosphonate drug on slowing down bone resorption which should allow for bone-forming cells the time needed to rebuild normal bone through bone remodeling. As shown in Fig 3, PC3 implantations treated with ZA revealed a bone-protective effect, with expedited healing of the surgical bone wound and progressive increases in overall bone density. MRI tumor volumes along with ADC values were quantified at day 21 post-treatment-initiation which revealed retardation of tumor growth in ZA-treated animals as compared to control animals (Fig 3A). Longitudinal CT scans on control and ZA-treated animals allowed for voxel-based PRM analysis to be accomplished for assessment of bone mineral changes due to the osteolytic processes associated with a growing PC3 tumor along with bone changes due to ZA intervention. Shown in Fig 3D are representative images for a control (top panels) and ZA-treated (bottom panels) mouse showing (from top to bottom) an isosurface, CT slice, PRM overlay, and PRM scatterplots from pre-treatment to 21 days post-treatment. The red regions represent voxels of increased bone mineral density versus loss of bone density depicted by blue voxels. Quantification of longitudinal changes in bone density was accomplished and summarized in Fig 3B and 3C. The PRM_HU+_ bar plot (Fig 3B) shows significantly higher volume of bone that increased in density after ZA-treatment as compared to control animals with growing PC3 tumors. The PRM_HU-_ bar plot (Fig 3C) showed minimal loss of bone occurred over time in the ZA-treated group as compared to progressively increasing bone loss in control PC3 implanted animals.

### PRM_CT_ Assessment of Osteolytic and Osteoblastic Responses to Metastatic Prostate Cancer

**Fig 4:**
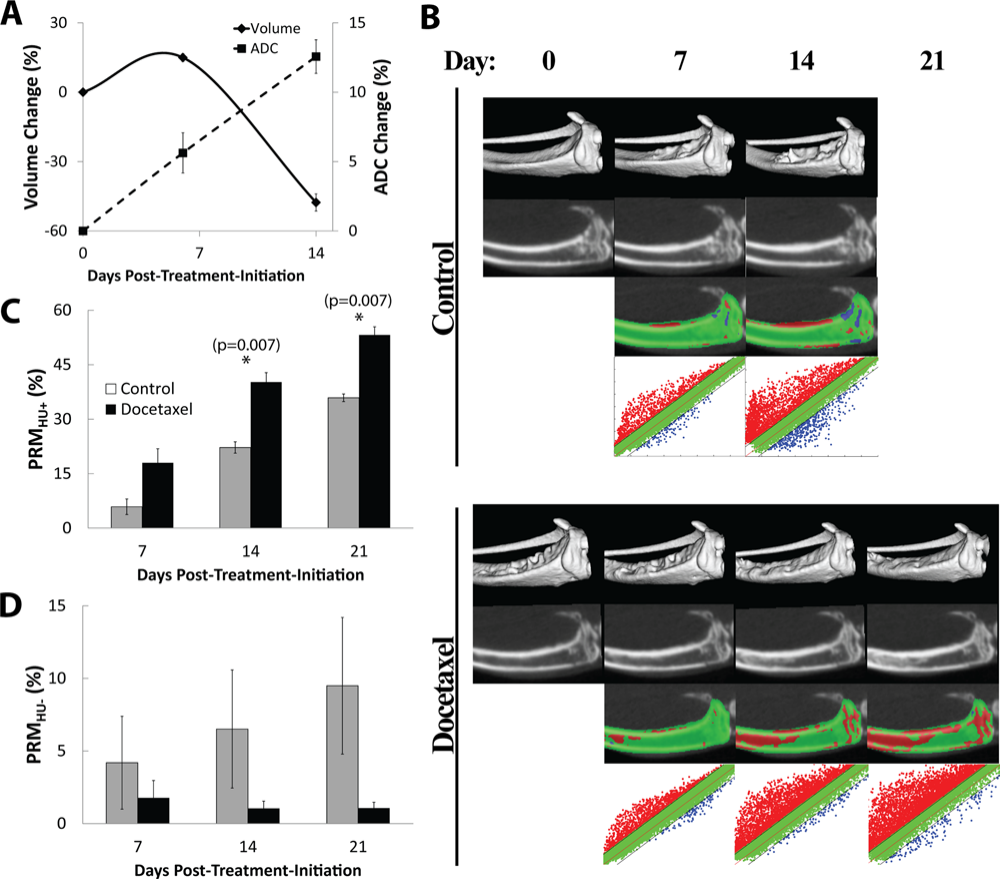
LAPC-9 tumors show slower mixed PRMHU+/- response with docetaxel treatment compared to PC3. (A) Time plots of tumor volume (solid line) and ADC (dashed line) show successful response to treatment (n = 3) as volume shrinkage and ADC increase. (B) PRM_HU+_ bar plot over time shows more bone density increase in the docetaxel-treated group compared to controls (n = 3), significant on days 14 and 21. (C) PRM_HU-_ bar plot over time shows very little bone loss in the treated group compared to elevated bone mineral loss in the controls (though not significant in this study). (D) Representative images for a control (top) and docetaxel-treated (bottom) mouse showing (from top to bottom) an isosurface, CT slice, PRM overlay, and PRM scatterplot from pre-treatment to 21 days post-treatment.

Docetaxel-treated LAPC-9 tumors were also evaluated against controls using PRM. The LAPC-9 tumor has been shown to have a significant osteoblastic effect when implanted in mice and PRM analysis revealed that there was in fact a slower and mixed PRM_HU_ response as compared to PC3 with docetaxel treatment. As shown in Fig 4A, plots of changes in tumor volume (solid line) and ADC (dashed line) revealed the effects of docetaxel-treatment on LAPC-9 as a decrease in tumor volume was associated with an early and increasing change in tumor ADC values. Shown in Fig 4D are representative images from a control animal (top panels) and a docetaxel-treated animal (bottom panels) showing (from top to bottom) an isosurface, CT slice, PRM overlay, and PRM scatterplot from pre-treatment to 21 days post-treatment. The untreated LAPC-9 tumors (Fig 4C) appeared to have less bone loss (PRM_HU-_) at day 21 compared to untreated PC3 animals (Fig 3C) indicating differential dynamics of bone loss and buildup between the two different tumor lines. As shown in Fig 4B, PRM_HU+_ bar plots over time reveal bone density increases occurred more in the docetaxel-treated group as compared to controls reaching significance by days 14 and 21. As shown in Fig 4C, PRM_HU-_ bar plots over time showed very little bone loss in the treated group compared to elevated bone mineral loss in the control animals (though not significant in this study).

**Fig 5:**
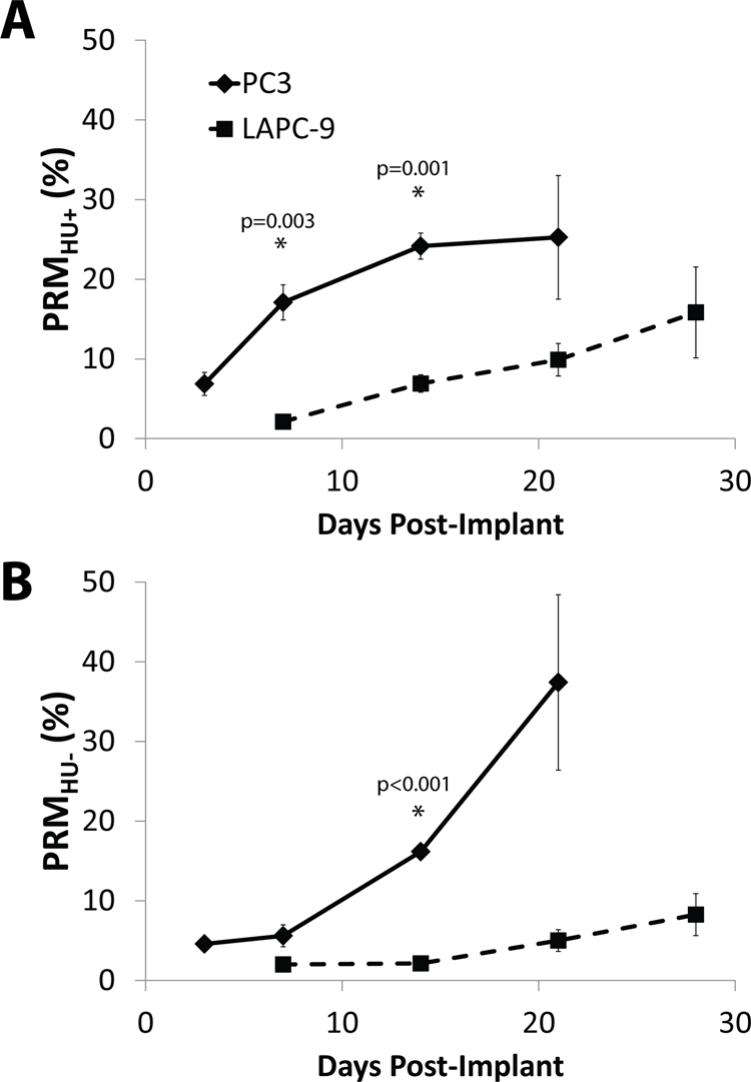
PRMHU plots over time post-implantation compare un-treated bone changes in PC3 (diamonds, solid line, n = 4) to LAPC-9 (squares, dashed line, n = 6) implants as quantified by (A) PRMHU+ and (B) PRMHU-.

Comparison between untreated PC3 and LAPC-9 tumor growth signatures via PRM analysis is shown in Fig 5. PRM_HU_ analysis for this comparison was performed using a µCT image acquired the day of implantation as the baseline, compared to the previous studies which used the day that treatments were initiated (pre-treatment) as baseline. In Fig 5A, PRM_HU+_ (gain of bone density) over time appeared to be greater in animals with PC3 tumors as compared to animals with LAPC-9 tumors. However, inspection of PRM color overlays for PC3 and LAPC-9 tumors in Figs 3D and 4D, respectively, shows that in fact most of the bone density increase was spatially associated with cortical bone expansion not local to the focal lesion reflecting structural changes associated with natural skeletal growth and compensation for the growing lesion. In contrast, clear differences in the effects of the two tumor lines on bone density loss is shown in Fig 5B wherein PRM_HU-_ values which reflect bone loss was significantly larger for untreated PC3 tumors versus animals with LAPC-9 tumors.

### Clinical PRM_ADC/HU_ Assessment of Metastatic Prostate Cancer Treatment Response

**Fig 6:**
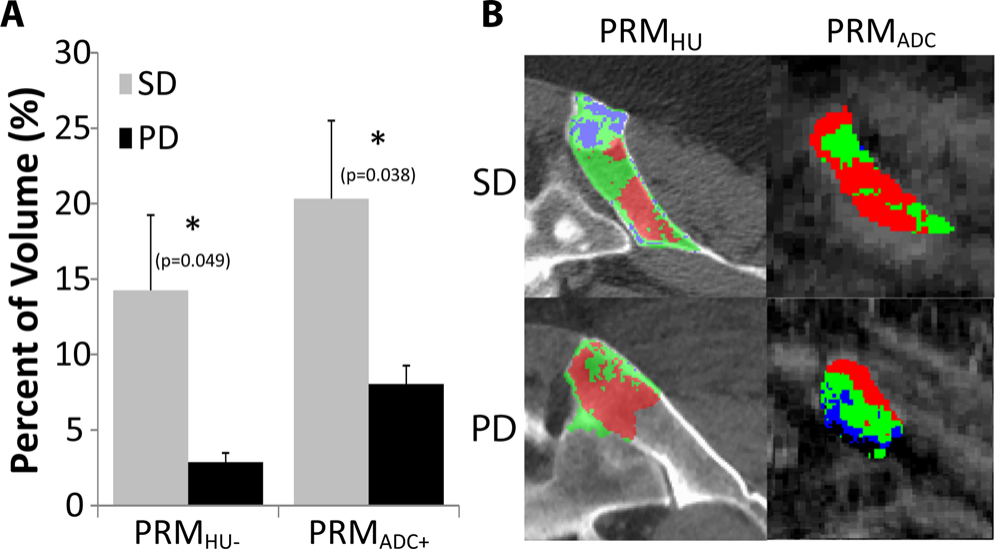
Results from clinical trial. (A) Representative PRM overlays show stable disease (top) and progressive disease (bottom) for PRMHU (left) and PRMADC+ (right). Blue represents regions of decreased value, red increased value, and green statistically unchanged value. (B) The bar plot shows significant differences (marked with *) between stable disease (SD, gray, n = 8) and progressive disease (PD, black, n = 4) groups in volume fractions of bone PRM results (labeled PRM_HU-_, volume fraction of decreased attenuation at about 10 weeks post-treatment) and DW-MRI (volume fraction of increased ADC at about 2 weeks post-treatment).

In the pilot clinical trial, patients with CRPC underwent both DW-MRI and CT scanning protocols. Of the 12 patients 8 were clinically determined to have stable disease (SD) and 4 to have disease progression (PD) by standard criteria at first disease assessment. DW-MRI PRM_ADC_ measurements were monitored per individual lesion on all 12 patients, resulting in 18 lesions for the SD group and 7 for the PD group. For CT-based PRM_HU_ measurements, 5 patients analyzed were SD (12 lesions) and 4 patients were PD (7 lesions). Representative PRM color overlays are presented in Fig 6A showing results for a SD patient (top) versus a PD patient (bottom) for PRM_HU_ (left) and PRM_ADC_ (right). In these panels, blue represents regions of decreased value, red increased value, and green statistically unchanged value. Shown in Fig 6B is a bar plot summarizing the imaging findings for patients with prostate cancer to the bone post-therapy. Follow-up DW-MR scans were obtained on average at 2.2±0.1 and 2.1±0.1 weeks’ post-therapy initiation for patients with stable disease (SD) and progressive disease (PD), respectively, and CT scans were obtained on average at 9.6±1.1 and 14.5±2.1 weeks post-therapy initiation for the SD and PD patient groups, respectively. Significant differences between SD and PD groups were found in both PRM_HU-_ and PRM_ADC+_. Early increases in tumor ADC quantified by PRM_ADC_ at 2 weeks post-treatment were positively correlated to treatment response based upon changes in PSA levels. Patients with clinically determined stable disease generally had a significantly greater volume fraction of their tumor with increased ADC values (17.2±3.9%) versus patients which were subsequently determined to have progressive disease (7.1±1.4%). Tumor-regional PRM_HU-_ volumes quantified by PRM_CT_ at approximately 9–12 weeks post-treatment were positively correlated to treatment response based upon changes in PSA levels. Patients with stable disease also generally had a significantly greater volume fraction of their tumor-regional VOI with reduced attenuation (13.2±4.2%) versus patients with progressive disease (2.5±0.6%).

## Discussion

Skeletal involvement remains the leading cause of morbidity and mortality in patients with prostate cancer. Bone scintigraphy with ^99m^Tc-labelled bisphosphonates is widely used to stage patients with bone metastasis [10]. The ability to optimize patient management in this population has been limited by difficulties in assessing response in bone metastasis based on bone scan imaging [7]. Quantification of treatment response of osseous lesions can be complicated by an inability to delineate accurate tumor dimensions due to a dynamic remodeling process occurring between osteoclasts and osteoblasts in an attempt to maintain bone homeostasis. Moreover, an increase in ^99m^Tc-labelled bisphosphonates by metastatic lesions may occur after initiation of systemic hormone or chemotherapy and is refered to as the flare phenomenon [51]. This phenomenon occurs due to bone remodeling in a skeletal metastasis as disregulation of processes involved with homeostasis can occur due to the presence of osteolytic, osteoblastic or mixed lesions contributing to an increased difficulty of image intrepretation.

The overall goal of this research study was to evaluate the voxel-based PRM biomarker for quantification of multi-modal images to assess the pathology and treatment effects of multiple tissues including tumor and stroma in the context of metastatic prostate cancer to the bone. Pathological correlation of loss of tumor cell density with increasing ADC values by DW-MR as well as loss of bone mineral density with decreasing in HU values in CT scans have been previously reported by our group [35,43]. Complex interactions between prostate cancer tumor cells and the host microenvironment and therapies targeting osseous metastases are dynamically maintained through a series of signaling pathways. The skeleton is remodeled continually through simultaneous resorption and formation of new bone. Significant effects on bone metabolism can be induced due to delivery of cancer treatments even in the local absence of a bone lesion known as cancer treatment-induced bone loss [52]. As the skeleton is the most common organ affected by metastatic cancer along with the site that produces the greatest patient morbidity, development of improved multi-modal imaging approaches which can provide for assessment of both the tumor and bone integrity may provide for new advances in the management of this patient population.

The objective of this study was to evaluate DW-MRI and CT as imaging biomarkers for quantification of growth progression and treatment response associated with changes in tumor cellularity (DW-MRI) and bone density (CT), respectively in metastatic cancer to the bone. We explored the capabilities of PRM voxel-based analysis of MRI and CT images to quantify and display changes in both tumor tissue and the bone stromal compartment in response to metastasis and treatment intervention. The hypothesis was that PRM may be useful for analyzing DW-MRI scans to provide for an early response biomarker and similiarly that PRM analysis of CT scans would provide insights on the impact of the tumor involvement and treatment on the stromal compartment.

Docetaxel plus prednisone is the standard first-line chemotherapy for patients with CRPC at time of study conduct. The study also allowed non-standard systemic therapy that has had reported efficacy in this disease setting. In previous work by our group [35], docetaxel therapy alone was shown to elicit a significant increase in ADC values for PC3 tumors. In this present study, MRI-determined PC3 tumor volumes showed that treatment with IR or docetaxel alone were both very effective in producing an overall growth delay but that the combination of the two interventions was much more effective than single agent therapies (Fig 2A). The PRM_ADC_ biomarker revealed that at day 7 post-treatment, a significant increase in tumor ADC values had occurred in the single therapy groups which were also found to be even higher in the combination treatment group (Fig 2B). Moreover, the data presented herein reveals that DW-MRI is capable of detecting early loss in tumor cellular density due to IR, docetaxel and radiochemotherapy in this bone metastasis model and that PRM analysis appeared to provide additional sensitivity for the combination therapy group over a simple histogram analysis (Fig 2B). The observed correlation of the magnitude of PRM_ADC_ changes as a quantitative imaging biomarker with outcome/tumor response in the setting of assessing therapeutic combinations may provide for improved screening opportunities in pre-clinical testing.

In this study, we also evaluated the ability to use PRM analysis of longitudinal CT scans to assess tumor-stromal interactions in bone metastasis models. Net loss of bone density was readily observed which increased over time in untreated PC3 animals. PC3 cells were used as they are known to generate an androgen independent osteolytic response [44,53]. PRM_HU_ analysis revealed that this approach could readily detect spatially and time-dependent loss of bone mineral density in this model (Fig 3) which was inhibited using anti-resorptive therapy (zoledronic acid). While the use of bisphosphonate treatment is considered standard of care for patient with metastic bone involvement, the mechanisms are not fully understood and the development of the PRM biomarkers allowing for simultaneous interrogation of the impact of these treatments on the tumor and stroma open up potentially new approches for obtaining information to further our understanding of the underlying dynamic changes which occur during therapy.

In an additional study, we used the LAPC-9 tumor line as it has been shown to have significant osteoblastic activity (Fig 4). This secondary tumor model was used to further evaluate the overall sensitivity of PRM analysis which revealed a slower and mixed PRM_HU_ progression in mice with LAPC-9 tumors as compared to PC3 mice (Fig 5). Where PC3 tumors resulted in a steady loss of bone seen as increasing PRM_HU-_, LAPC-9 tumors resulted in progressive increases in both PRM_HU+_ and PRM_HU-_. In fact, bone loss was found to be significantly higher in untreated PC3 tumors versus animals with LAPC-9 tumors. Significantly higher PRM_HU+_ seen in the PC3 tumors was attributed to the need for the remaining healthy bone to compensate for the loss of bone structure near the tumor. The PRM analysis was able to detect and quantify osteolytic versus osteoblastic lesions.

In the pilot clinical trial, we used both DW-MRI and CT scanning protocols to evaluate their ability to detect changes in patients with CRPC. While patient numbers were relatively small, the volume fraction of the tissue that had a significant decrease in HU value (PRM_HU-_) was found to be greater in the SD group versus the PD patient group. This may indicate bone re-normalization in patients who responded to therapy. Although lesion phenotyping was not performed in this study, regions of the metastases generally seemed to appear sclerotic on CT images, which agree with the hypothesis that a decrease in attenuation in these regions may indicate progress toward a normal state and positive treatment outcome. The use of PRM_HU_ in the context of monitoring changes due to bone metastases and treatment intervention appears promising and further studies based upon these novel findings are warranted. Moreover, DW-MRI scans (PRM_ADC_) at 2 weeks post-treatment revealed that early increases in tumor ADC values could be detected which were positively correlated to a subsequent positive clinical response. The fact that patients with SD were shown to have a significantly greater volume fraction of their tumors with increased ADC values versus PD patients also reveals the potential for using PRM_ADC_ as an imaging biomarker for early treatment stratification of patients with metastatic prostate cancer to the bone. These results support earlier findings in hormone naïve patients which showed similar increased PRM_ADC_ values in therapeutically responsive patients [27,36]. Moreover, methodologies for assessment of response in disseminated skeletal metastases using whole body DW-MR have also recently emerged [54,55].

Overall, this study revealed that the PRM imaging biomarker as applied to DW-MRI and CT scans was able to provide for quantitative assessment of tumor-stroma (bone) interactions which were specific to overall treatment outcome with similar findings in both the mouse and human studies. Further development and evaluation of DW-MRI and methods for quantification and visualization of response such as PRM as a noninvasive quantitative imaging biomarker are warranted to more fully explore the clinical impact of this approach.

## Supporting Information

S1 CONSORT Checklist TREND statement checklist for nonrandomized controlled trial. (PDF)

S1 Protocol Protocol for Early Assessment of Treatment Response using Functional Diffusion Mapping (PDF)

## References

[1] Onukwugha E, Yong C, Mullins CD, Seal B, McNally D, Hussain A. Skeletal-related events and mortality among older men with advanced prostate cancer. J Geriatr Oncol. 2014. 10.1016/j.jgo.2014.03.002 24726866

[2] Saman DM, Lemieux AM, Nawal Lutfiyya M, Lipsky MS. A review of the current epidemiology and treatment options for prostate cancer. Dis Mon. 2014; 60: 150–154. 10.1016/j.disamonth.2014.02.003 24726082

[3] Carlin BI, Andriole GL. The natural history, skeletal complications, and management of bone metastases in patients with prostate carcinoma. Cancer. 2000; 88: 2989–2994. 10898342 10.1002/1097-0142(20000615)88:12+<2989::aid-cncr14>3.3.co;2-h

[4] Tannock IF, de Wit R, Berry WR, Horti J, Pluzanska A, Chi KN, et al Docetaxel plus prednisone or mitoxantrone plus prednisone for advanced prostate cancer. N Engl J Med. 2004; 351: 1502–1512. 15470213 10.1056/NEJMoa040720

[5] Bubendorf L, Schopfer A, Wagner U, Sauter G, Moch H, Willi N, et al Metastatic patterns of prostate cancer: an autopsy study of 1,589 patients. Hum Pathol. 2000; 31: 578–583. 10836297 10.1053/hp.2000.6698

[6] Taichman RS, Loberg RD, Mehra R, Pienta KJ. The evolving biology and treatment of prostate cancer. J Clin Invest. 2007; 117: 2351–2361. 17786228 10.1172/JCI31791 PMC1952634

[7] Eisenhauer E, Therasse P, Bogaerts J, Schwartz L, Sargent D, Ford R, et al New response evaluation criteria in solid tumours: revised RECIST guideline (version 1.1). European journal of cancer. 2009; 45: 228–247. 10.1016/j.ejca.2008.10.026 19097774

[8] Miller TT. Bone tumors and tumorlike conditions: analysis with conventional radiography. Radiology. 2008; 246: 662–674. 10.1148/radiol.2463061038 18223119

[9] Abdel-Dayem HM. The role of nuclear medicine in primary bone and soft tissue tumors. Semin Nucl Med. 1997; 27: 355–363. 9364645 10.1016/s0001-2998(97)80008-6

[10] Hamaoka T, Madewell JE, Podoloff DA, Hortobagyi GN, Ueno NT. Bone imaging in metastatic breast cancer. J Clin Oncol. 2004; 22: 2942–2953. 15254062 10.1200/JCO.2004.08.181

[11] Wondergem M, van der Zant FM, van der Ploeg T, Knol RJ. A literature review of 18F-fluoride PET/CT and 18F-choline or 11C-choline PET/CT for detection of bone metastases in patients with prostate cancer. Nucl Med Commun. 2013; 34: 935–945. 10.1097/MNM.0b013e328364918a 23903557

[12] Glendenning J, Cook G. Imaging breast cancer bone metastases: current status and future directions. Semin Nucl Med. 2013; 43: 317–323. 10.1053/j.semnuclmed.2013.02.002 23725993

[13] Costelloe CM, Chuang HH, Madewell JE, Ueno NT. Cancer Response Criteria and Bone Metastases: RECIST 1.1, MDA and PERCIST. J Cancer. 2010; 1: 80–92. 20842228 10.7150/jca.1.80 PMC2938069

[14] Kransdorf MJ, Bridges MD. Current developments and recent advances in musculoskeletal tumor imaging. Semin Musculoskelet Radiol. 2013; 17: 145–155. 10.1055/s-0033-1343070 23673546

[15] Le Bihan D. The 'wet mind': water and functional neuroimaging. Phys Med Biol. 2007; 52: R57–90. 17374909 10.1088/0031-9155/52/7/R02

[16] Le Bihan D. Apparent diffusion coefficient and beyond: what diffusion MR imaging can tell us about tissue structure. Radiology. 2013; 268: 318–322. 10.1148/radiol.13130420 23882093

[17] Moseley ME, Cohen Y, Mintorovitch J, Chileuitt L, Shimizu H, Kucharczyk J, et al Early detection of regional cerebral ischemia in cats: comparison of diffusion- and T2-weighted MRI and spectroscopy. Magn Reson Med. 1990; 14: 330–346. 2345513 10.1002/mrm.1910140218

[18] Padhani AR, Liu G, Koh DM, Chenevert TL, Thoeny HC, Takahara T, et al Diffusion-weighted magnetic resonance imaging as a cancer biomarker: consensus and recommendations. Neoplasia. 2009; 11: 102–125. 19186405 10.1593/neo.81328 PMC2631136

[19] Ross BD CT, Kim B, Ben-Yoseph O. Magnetic resonance imaging and spectroscopy: application to experimental neuro-oncology. Q Magn Reson Biol Med. 1994; 1: 89–106. 26550608 PMC4634890

[20] Thoeny HC, Ross BD. Predicting and monitoring cancer treatment response with diffusion-weighted MRI. J Magn Reson Imaging. 2010; 32: 2–16. 10.1002/jmri.22167 20575076 PMC2918419

[21] Bains LJ, Zweifel M, Thoeny HC. Therapy response with diffusion MRI: an update. Cancer Imaging. 2012; 12: 395–402. 23022595 10.1102/1470-7330.2012.9047 PMC3460562

[22] Galban S, Brisset JC, Rehemtulla A, Chenevert TL, Ross BD, Galban CJ. Diffusion-weighted MRI for assessment of early cancer treatment response. Curr Pharm Biotechnol. 2010; 11: 701–708. 20504274 10.2174/138920110792246627 PMC4003912

[23] Williams TM, Galban S, Li F, Heist KA, Galban CJ, Lawrence TS, et al DW-MRI as a Predictive Biomarker of Radiosensitization of GBM through Targeted Inhibition of Checkpoint Kinases. Transl Oncol. 2013; 6: 133–142. 23544166 10.1593/tlo.13214 PMC3610547

[24] Hamstra DA, Galban CJ, Meyer CR, Johnson TD, Sundgren PC, Tsien C, et al Functional diffusion map as an early imaging biomarker for high-grade glioma: correlation with conventional radiologic response and overall survival. J Clin Oncol. 2008; 26: 3387–3394. 10.1200/JCO.2007.15.2363 18541899 PMC3266717

[25] Moffat BA, Chenevert TL, Lawrence TS, Meyer CR, Johnson TD, Dong Q, et al Functional diffusion map: a noninvasive MRI biomarker for early stratification of clinical brain tumor response. Proc Natl Acad Sci U S A. 2005; 102: 5524–5529. 15805192 10.1073/pnas.0501532102 PMC555936

[26] Moffat BA, Chenevert TL, Meyer CR, McKeever PE, Hall DE, Hoff BA, et al The functional diffusion map: an imaging biomarker for the early prediction of cancer treatment outcome. Neoplasia. 2006; 8: 259–267. 16756718 10.1593/neo.05844 PMC1600674

[27] Lee KC, Bradley DA, Hussain M, Meyer CR, Chenevert TL, Jacobson JA, et al A feasibility study evaluating the functional diffusion map as a predictive imaging biomarker for detection of treatment response in a patient with metastatic prostate cancer to the bone. Neoplasia. 2007; 9: 1003–1011. 18084607 10.1593/neo.07954 PMC2134897

[28] Lemasson B, Galbán CJ, Boes JL, Li Y, Zhu Y, Heist KA, et al Diffusion-Weighted MRI as a Biomarker of Tumor Radiation Treatment Response Heterogeneity: A Comparative Study of Whole-Volume Histogram Analysis versus Voxel-Based Functional Diffusion Map Analysis. Translational oncology. 2013; 6: 554 24151536 10.1593/tlo.13532 PMC3799198

[29] Ma B, Meyer CR, Pickles MD, Chenevert TL, Bland PH, Galbán CJ, et al Voxel-by-voxel functional diffusion mapping for early evaluation of breast cancer treatment; 2009 Springer pp. 276–287. 10.1007/978-3-642-02498-6_23 PMC2804941 19694270

[30] Ellingson BM, Cloughesy TF, Lai A, Mischel PS, Nghiemphu PL, Lalezari S, et al Graded functional diffusion map-defined characteristics of apparent diffusion coefficients predict overall survival in recurrent glioblastoma treated with bevacizumab. Neuro Oncol. 2011; 13: 1151–1161. 10.1093/neuonc/nor079 21856685 PMC3177656

[31] Ellingson BM, Cloughesy TF, Lai A, Nghiemphu PL, Pope WB. Nonlinear registration of diffusion-weighted images improves clinical sensitivity of functional diffusion maps in recurrent glioblastoma treated with bevacizumab. Magn Reson Med. 2012; 67: 237–245. 10.1002/mrm.23003 21702063

[32] Ellingson BM, Cloughesy TF, Zaw T, Lai A, Nghiemphu PL, Harris R, et al Functional diffusion maps (fDMs) evaluated before and after radiochemotherapy predict progression-free and overall survival in newly diagnosed glioblastoma. Neuro Oncol. 2012; 14: 333–343. 10.1093/neuonc/nor220 22270220 PMC3280805

[33] Ellingson BM, Malkin MG, Rand SD, Connelly JM, Quinsey C, LaViolette PS, et al Validation of functional diffusion maps (fDMs) as a biomarker for human glioma cellularity. J Magn Reson Imaging. 2010; 31: 538–548. 10.1002/jmri.22068 20187195 PMC2903058

[34] Hiramatsu R, Kawabata S, Furuse M, Miyatake S, Kuroiwa T. Identification of early and distinct glioblastoma response patterns treated by boron neutron capture therapy not predicted by standard radiographic assessment using functional diffusion map. Radiat Oncol. 2013; 8: 192 10.1186/1748-717X-8-192 23915330 PMC3751226

[35] Lee KC, Sud S, Meyer CR, Moffat BA, Chenevert TL, Rehemtulla A, et al An imaging biomarker of early treatment response in prostate cancer that has metastasized to the bone. Cancer Res. 2007; 67: 3524–3528. 17440058 10.1158/0008-5472.CAN-06-4236

[36] Reischauer C, Froehlich JM, Koh DM, Graf N, Padevit C, John H, et al Bone metastases from prostate cancer: assessing treatment response by using diffusion-weighted imaging and functional diffusion maps—initial observations. Radiology. 2010; 257: 523–531. 10.1148/radiol.10092469 20829534

[37] Rozel S, Galban CJ, Nicolay K, Lee KC, Sud S, Neeley C, et al Synergy between anti-CCL2 and docetaxel as determined by DW-MRI in a metastatic bone cancer model. J Cell Biochem. 2009; 107: 58–64. 10.1002/jcb.22056 19259948 PMC4293017

[38] Galban CJ, Chenevert TL, Meyer CR, Tsien C, Lawrence TS, Hamstra DA, et al The parametric response map is an imaging biomarker for early cancer treatment outcome. Nat Med. 2009; 15: 572–576. 10.1038/nm.1919 19377487 PMC3307223

[39] Galban CJ, Mukherji SK, Chenevert TL, Meyer CR, Hamstra DA, Bland PH, et al A feasibility study of parametric response map analysis of diffusion-weighted magnetic resonance imaging scans of head and neck cancer patients for providing early detection of therapeutic efficacy. Transl Oncol. 2009; 2: 184–190. 19701503 10.1593/tlo.09175 PMC2730136

[40] Chiba Y, Kinoshita M, Okita Y, Tsuboi A, Isohashi K, Kagawa N, et al Use of (11)C-methionine PET parametric response map for monitoring WT1 immunotherapy response in recurrent malignant glioma. J Neurosurg. 2012; 116: 835–842. 10.3171/2011.12.JNS111255 22242671

[41] Tsien C, Galban CJ, Chenevert TL, Johnson TD, Hamstra DA, Sundgren PC, et al Parametric response map as an imaging biomarker to distinguish progression from pseudoprogression in high-grade glioma. J Clin Oncol. 2010; 28: 2293–2299. 10.1200/JCO.2009.25.3971 20368564 PMC2860441

[42] Harris RJ, Cloughesy TF, Pope WB, Nghiemphu PL, Lai A, Zaw T, et al 18F-FDOPA and 18F-FLT positron emission tomography parametric response maps predict response in recurrent malignant gliomas treated with bevacizumab. Neuro Oncol. 2012; 14: 1079–1089. 10.1093/neuonc/nos141 22711609 PMC3408264

[43] Hoff BA, Kozloff KM, Boes JL, Brisset JC, Galban S, Van Poznak CH, et al Parametric response mapping of CT images provides early detection of local bone loss in a rat model of osteoporosis. Bone. 2012; 51: 78–84. 10.1016/j.bone.2012.04.005 22542461 PMC3371150

[44] Kaighn ME, Narayan KS, Ohnuki Y, Lechner JF, Jones LW. Establishment and characterization of a human prostatic carcinoma cell line (PC-3). Invest Urol. 1979; 17: 16–23. 447482

[45] Moffat BA, Hall DE, Stojanovska J, McConville PJ, Moody JB, Chenevert TL, et al Diffusion imaging for evaluation of tumor therapies in preclinical animal models. MAGMA. 2004; 17: 249–259. 15580371 10.1007/s10334-004-0079-z

[46] Craft N, Chhor C, Tran C, Belldegrun A, DeKernion J, Witte ON, et al Evidence for clonal outgrowth of androgen-independent prostate cancer cells from androgen-dependent tumors through a two-step process. Cancer Res. 1999; 59: 5030–5036. 10519419

[47] Klein KA, Reiter RE, Redula J, Moradi H, Zhu XL, Brothman AR, et al Progression of metastatic human prostate cancer to androgen independence in immunodeficient SCID mice. Nat Med. 1997; 3: 402–408. 9095173 10.1038/nm0497-402

[48] Nickerson T, Chang F, Lorimer D, Smeekens SP, Sawyers CL, Pollak M. In vivo progression of LAPC-9 and LNCaP prostate cancer models to androgen independence is associated with increased expression of insulin-like growth factor I (IGF-I) and IGF-I receptor (IGF-IR). Cancer Res. 2001; 61: 6276–6280. 11507082

[49] Hoff BA, Chenevert TL, Bhojani MS, Kwee TC, Rehemtulla A, Le Bihan D, et al Assessment of multiexponential diffusion features as MRI cancer therapy response metrics. Magn Reson Med. 2010; 64: 1499–1509. 10.1002/mrm.22507 20860004 PMC2965786

[50] Meyer CR, Boes JL, Kim B, Bland PH, Zasadny KR, Kison PV, et al Demonstration of accuracy and clinical versatility of mutual information for automatic multimodality image fusion using affine and thin-plate spline warped geometric deformations. Med Image Anal. 1997; 1: 195–206. 9873906 10.1016/s1361-8415(97)85010-4

[51] Cook GJ, Venkitaraman R, Sohaib AS, Lewington VJ, Chua SC, Huddart RA, et al The diagnostic utility of the flare phenomenon on bone scintigraphy in staging prostate cancer. Eur J Nucl Med Mol Imaging. 2011; 38: 7–13. 10.1007/s00259-010-1576-0 20697891

[52] Esposito M, Kang Y. Targeting tumor-stromal interactions in bone metastasis. Pharmacol Ther. 2014; 141: 222–233. 10.1016/j.pharmthera.2013.10.006 24140083 PMC3947254

[53] Corey E, Quinn JE, Bladou F, Brown LG, Roudier MP, Brown JM, et al Establishment and characterization of osseous prostate cancer models: intra-tibial injection of human prostate cancer cells. Prostate. 2002; 52: 20–33. 11992617 10.1002/pros.10091

[54] Blackledge MD, Collins DJ, Tunariu N, Orton MR, Padhani AR, Leach MO, et al Assessment of treatment response by total tumor volume and global apparent diffusion coefficient using diffusion-weighted MRI in patients with metastatic bone disease: a feasibility study. PLoS One. 2014; 9: e91779 10.1371/journal.pone.0091779 24710083 PMC3977851

[55] Padhani AR, Makris A, Gall P, Collins DJ, Tunariu N, de Bono JS. Therapy monitoring of skeletal metastases with whole-body diffusion MRI. J Magn Reson Imaging. 2014; 39: 1049–1078. 10.1002/jmri.24548 24510426

