# Supplementary material for: Integrated Multimodal Imaging of Dynamic Bone-Tumor Alterations Associated with Metastatic Prostate Cancer
Source: PLoS One. 2015 Apr 10;10(4):e0123877. doi: 10.1371/journal.pone.0123877 (PMC4393258; doi:10.1371/journal.pone.0123877)
Supplement: S1 Protocol — (PDF) [file pone.0123877.s002.pdf]

Early Assessment of Treatment Response using Functional Diffusion Mapping

Co-Principal Investigators:

Maha Hussain, M.D., FACP
7314 Cancer Center, SPG 5946
1500 E. Medical Center Drive
Ann Arbor, MI, 48109-5946
Telephone: 734-936-8906
Fax: 734-615-2719
Email: mahahuss@med.umich.edu

Co-Investigators:

Medical Oncology: Kathleen Cooney, M.D.
Kenneth Pienta, M.D.
David Smith, M.D.
Radiology: Thomas Chenevert, Ph.D.
Jon Jacobson, M.D.
Craig Galbán, Ph.D.
Charles Meyer, Ph.D.
Brian Ross, Ph.D.
Translational Studies: Evan Keller, D.V.M., Ph.D.
Biostatistics: Timothy Johnson, Ph.D.

Responsible Research Nurse:

Tamara Huebner, RN, BSN, OCN
B1-245 Cancer Center, SPG 5913
1500 E. Medical Center Dr.

Ann Arbor, MI 48109-5913
Tel: (734) 763-4992
Email: thuebner@med.umich.edu

Responsible Data Manager:

Amie Anderson, Clinical Subjects Coordinator
Clinical Trials Office
North Campus Research Complex
2800 Plymouth Road Building 300
Ann Arbor, MI 48105-2967
Tel: (734) 615-1749 Fax: (734) 232-0744
Email: acander@med.umich.edu

Protocol version: 4.5.08 (Amendment 2)
8.03.10 (Amendment 3)

Table of Contents

Schema.. 4

1. Objectives...5

2. Background and Significance..5
- **2.1. Metastatic Hormone Refractory Prostate Cancer**..5
- **2.2. Current Assessment of Bone Lesions in Prostate Cancer Clinical Trials**..........5
- **2.3. Diffusion MRI to Monitor Anti-Cancer Therapy**……………………….. …. .6
- **2.4. Biochemical Markers of Bone Turnover to Assess Treatment Response**……..8
- **2.5. Markers of Apoptosis to Assess Treatment Response**…..…………………….. 8
- **2.6. Study Rationale**…………………………………………………………………9

3. Patient Selection………………………………………………………………… 10
- . **3.1. Inclusion Criteria**…………………………………………………............... 10
- **3.2. Exclusion Criteria**……………………………………………………….. 10
- **3.3. Inclusion of Women and Minorities**…………………………………………… 10

4. Registration………………………………………………………………........... 11

5. Protocol Methods………………………………………………………….......... 11
- **5.1. Imaging Protocol**………………………………………………………………… 11
- **5.2. Correlative Studies Procedures and Methods**………………………………… 14

6. Duration of Study……………………………………………………………......... 15

7. Criteria for Removal from Study…………………………………………………… 15

8. Statistical Considerations…………………………………………………………… 16
- **8.1. Study Design/Endpoints**…..……………………………………………………16
- **8.2. Sample Size/Accrual Rate**………..………………………………………......... 17

9. Study Calendar………………………………………………………………………… 17

10. Measurement of Effect…………………………………………………….............. 18
- **10.1. Outcomes Based on Post-Therapy PSA Changes**……………………………….. 18
- **10.2. Changes Based on Measurable Disease**……………………………………….. 18
- **10.3 Outcomes Based on Radionucleotide Bone Scans**……………………………. 18
- **10.4 Definition of Progression Based on Pain**……………………………………… 19

11. Regulatory and Reporting Requirements………………………………………………. 19

12. Data Management and Monitoring………………………………………......... 20
- **12.1. Data Collection/Entry**…………………………………………………….. 20
- **12.2. Data Submissions**………………………………………………………….. 20

12.3. Data Security and Confidentiality……………………………………………….. 20
12.4. Quality Assurance/Data Safety and Monitoring……………………….......... 20

13. References……………………………………………………………………….......... 22

Appendix A. MRI Checklist………………………………………………………………… 27

Appendix B. Guidelines for MRI Compatibility of Surgical Hardware/Implants………. 28

Appendix C. Specimen Collection Form…………………………………………………… 29

Schema

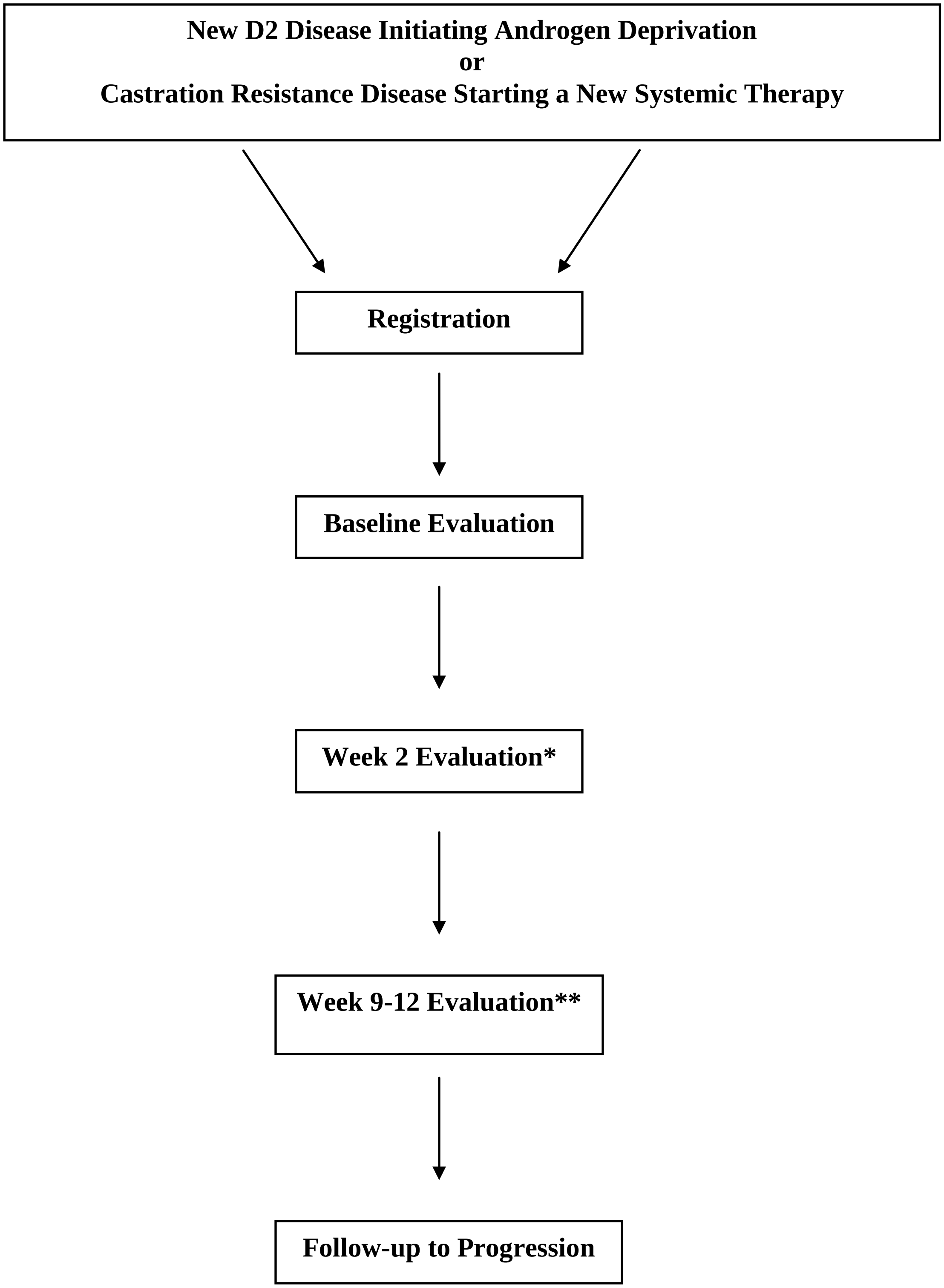

*First assessment by MRI: during week 2 (2nd week of cycle 1 for men being treated with chemotherapy).
**Second assessment: between weeks 9-12 (after 3-4 cycles for men being treated with non hormonal therapy).

1. OBJECTIVES

1.1 Primary Objective

- To identify the changes in diffusion MRI that correlate with response to therapy.

1.2 Secondary Objective

- Correlate changes in serum CK18Asp396 (a marker of apoptosis) with functional diffusion maps and treatment response.
- Correlate changes in biochemical markers of bone turnover with functional diffusion maps and treatment response.

2. BACKGROUND

2.1 Metastatic Hormone Refractory Prostate Cancer

It is estimated that in 2006, 27,350 men in the United States will die of castration resistant prostate cancer.[1] Based on prolongation of survival demonstrated in two randomized phase III trials, systemic chemotherapy with docetaxel has been established as standard of care for treatment of this patient population.[2, 3] However, improvement in survival is modest and docetaxel-based therapy has no curative potential in this setting.[2, 3] Thus, investigation of new systemic therapy is a medical necessity.

One of the recognized difficulties in investigating new systemic treatments for metastatic prostate cancer is the inability to accurately and objectively assess response to therapy. This results from the fact that bone is the predominant site of disease in 85-90% of men,[2, 3] and in the majority of cases, the only site of metastatic disease. Accurate assessment of baseline bone involvement and response in bone to treatment is difficult using standard imaging tools. Thus, currently, bone disease is considered unmeasurable by RECIST criteria. This has significantly hampered progress in therapy development for prostate cancer.

To overcome these limitations, PSA alterations have been explored as a potential response measure. Although a decline in PSA of at least 50% in response to therapy has become widely accepted as a screening tool for anti-tumor effect,[4] the use of PSA endpoints have not been prospectively validated in phase III trials as a surrogate measure of survival. Furthermore, as the therapeutic armamentarium available has increased to include novel agents and targeted therapy with mechanisms of action that differ from classic cytotoxic therapy, changes in PSA have proven to be an inadequate measure of meaningful clinical response.[5, 6] Thus, technology that could reliably and accurately measure anti-tumor effect in bone would provide a significant advance in prostate cancer treatment as it will facilitate the timely evaluation of new agents in clinical trials.

2.2 Current Assessment of Bone Lesions in Prostate Cancer Clinical Trials

The consensus recommendations published by the PSA Working Group in 1999 maintained the clinical standard of using radionucleotide bone scans to assess bone metastasis and response of bone lesions.[4] Although considered to be the most practical screening technique

for assessing the entire skeleton for skeletal metastases, it is well recognized that bone scanning lacks the sensitivity and specificity needed to accurately distinguish metastatic lesions from areas of abnormal radionuclide uptake due to inflammation, degeneration, or trauma and to measure therapeutic response. These limitations stem from the fact that bone scanning measures only metabolic activity and does not evaluate structural integrity of bone. Scintigraphic flare, a well recognized clinical phenomenon describing apparent progression of individual lesions on bone scan despite clinical improvement[7-11] is one important example. In prostate cancer, scintigraphic flare has been documented in up to 23% of scans.[12] Because scintigraphic flare is observed approximately 3 months after the initiation of therapy for bone metastasis, it may not be possible to evaluate response to therapy accurately until 6 months after therapy.[13]

2.3 Diffusion MRI to Monitor Anti-Cancer Therapy

The use of diffusion MRI technology to assess response to anti-cancer therapy is based on detecting changes in the Brownian motion of water. The diffusion of water in tissue is strongly affected by the viscosity of intra- and extra-cellular fluids, membrane permeability, active transport and flow, and directionality of cellular structures that enhance or impede its mobility and thus can be considered to be a quantifiable biophysical measurement of the tumor microenvironment. During the course of successful therapeutic intervention, changes in cellular structure occur which precede macroscopic changes such as decreases in overall tumor volume. These changes are detectable as a quantifiable change in the apparent diffusion coefficient of tumor water (ADC). As individual cancer cells are damaged in response to a therapeutic intervention, the integrity of cell membranes is compromised resulting in a decrease in the amount of restrictive barriers to water diffusion. Thus, the water mobility within a tumor will increase over time following effective treatment as represented by an increase in the ADC with the magnitude of the change related to the effectiveness of the therapy. This principal has been successfully applied preclinically in studies assessing response to a variety of anti-cancer agents including: cytotoxic and cytostatic therapies, radiation therapy, and gene therapy.[14-25] However, the response of ADC to therapy in the clinical setting was found to be more complex due to heterogeneity observed within human tumors.[26, 27] To this end, functional diffusion map (fDM) was developed as a statistical approach for segmenting tumors based on a defined threshold of ADC change following therapy.[27]

Results from patients with primary malignant brain tumors have been analyzed using the fDM approach revealing that the volume of fDM response had a strong correlation with the overall clinical response based on the World Health Organization response criteria.[27] In a more recent study,[28] patients with grade III/IV gliomas were analyzed using fDM, revealing that fDM could be used to stratify patients as responsive or nonresponsive to therapy in as early as 3 weeks into a 6-7-week fractionated therapy schedule. In this study, patients identified by fDM as nonresponsive had significantly poorer survival and time-to progression than patients identified as responsive.[28] Based on the promising results of these studies, further investigation of fDM to serve as an early biomarker of treatment response in other tumor types is being investigated. In a feasibility study using fDM to monitor treatment response in head and neck cancer, the distribution of ADC values moved to higher values

midway through the first cycle of treatment and that the increase in mean ADC appeared to be higher for CR lesions than PR lesions (personal communication) proving fDM can be useful in evaluating treatment response outside of the brain.

To date, fDM has not been investigated clinically as a means of assessing treatment response in bone. We have utilized a preclinical model to investigate the feasibility of this technology to detect changes in bone metastases in response to therapy. Utilizing PC3 prostate cancer xenografts with confirmed bone metastases, we have shown a correlation between changes in fDM and changes in bioluminescence imaging (a sensitive imaging modality that allows for rapid screening of mice to identify tumor location, extent of disease, and monitor response to treatment) in response to docetaxel treatment with changes seen by day 7 (unpublished results) proving fDM of bone metastases is feasible. In addition, diffusion MRI imaging of human bone has proven practical. Utilizing differences in ADC, investigators have been able to differentiate benign from pathologic vertebral compression fractures and malignant from infectious processes[29-32] and to assess response to radiation therapy in patients with metastatic disease of the spine.

Figure 1.

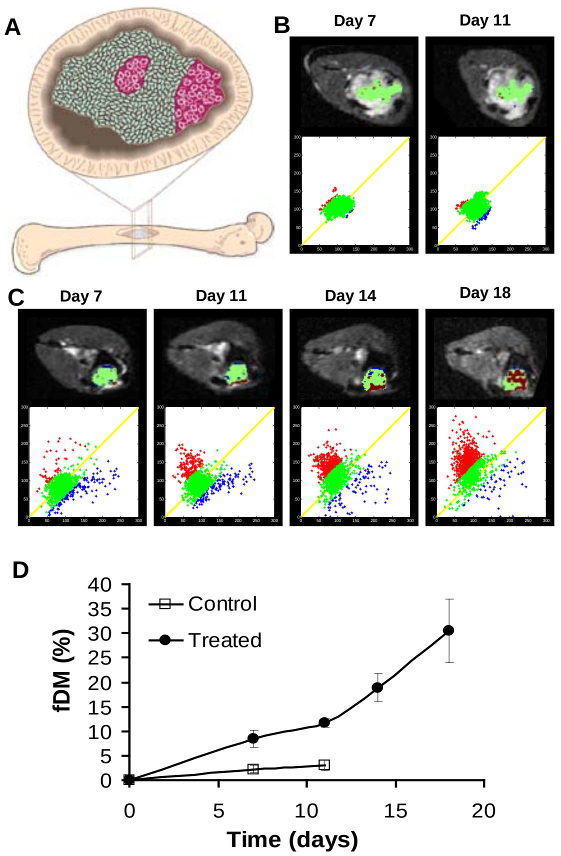

(A.) Schematic representation of changes detected in bone. (B.) Untreated and (C.) Docetaxel - treated representative fDM data obtained from our prostate cancer xenografts. Image datasets from baseline and after therapy are coregistered and analyzed to yield fDM. These maps present a color overlay of therapeutic induced ADC changes within tumor where different regions within the same tumor are stratified based on increasing (red), decreasing (blue), or stable (green) ADC. (D.) Plot of mean percentage changes in fDM over time. Treatment-induced cell kill leads to decreased membrane integrity and thus increase in ADC. Thus a greater increase in % change of fDM is representative of greater treatment response.

2.4 Biochemical Markers of Bone Turnover to Assess Treatment Response

Biochemical markers of bone turnover can be classified as markers of bone resorption and bone formation reflecting osteoclastic and osteoblastic activity respectively. Originally used to evaluate treatment of metabolic bone disease, over the last several years, the potential utility of bone turnover to measure efficacy of bone targeted therapy in cancer has been realized. [33-35] Given that bone is the predominant site and often only site of disease in 85-90% of men with metastatic prostate cancer,[2, 3] the use of bone turnover makers to monitor therapy has been of particular interest in this disease. Although in prostate cancer, bone metastases are mostly osteoblastic in nature, it is well recognized that prostate cancer metastases result from a heterogeneous mixture of osetoblastic and osteolytic lesions.[36-39] It has been shown that osteoblastic metastases form on trabecular bone at sites of previous osteoclast resorption and that such resorption is required for subsequent osteoblastic bone formation.[40, 41] These findings suggest that prostate cancer induces bone production through an overall increase in bone remodeling;[42-44] thus investigation of markers of osteoblastic and osteoclastic activity are relevant in evaluating baseline disease activity and monitoring therapeutic response.

In prostate cancer, investigation of bone turnover markers has largely been limited to studies of bisphosphonate use. In this setting, elevated markers of osteolytic activity (N-telopeptide) and osteoblastic activity (bone-specific alkaline phosphatase) have been associated with adverse clinical outcomes including shorter time to skeletal events, disease progression and death.[33-35] Additionally, a correlation between baseline values of N-telopeptide, bone-specific alkaline phosphatase, PSA and number of bone lesions have also been shown[35, 45] suggesting baseline markers of osteolytic and osteoblastic activity correlate with tumor burden. There is little data published on the effect of chemotherapy on bone makers. This is in large part secondary to the ability of PSA to serve as a surrogate of response in traditional cytoxic therapies. However, as the potential treatment armentarium available has increased to include targeted therapeutics that may not be cytotoxic, standard clinical endpoints of efficacy such as tumor response rates may not be as applicable. Therefore the role of bone turnover markers is again being investigated to aide in assessing treatment response, especially in bone targeted therapy.[5, 6, 46, 47] Results from phase II and III studies of atrasentan, an investigational agent that inhibits the endothelin-A receptor resulting in decreased osteoblast activity, has shown no significant responses as assessed by classical measures yet it appeared to result in an increase in time to progression in patients with bone meatstatses. [5, 6, 40] Investigation of bone markers in these studies has also demonstrated suppression of biochemical markers of bone turnover supporting the potential role of bone turnover markers in investigating efficacy of novel agents.

2.5 Markers of Apoptosis to Assess Treatment Response

Apoptosis is a unique physiological mechanism leading to cell death.[48, 49] Many established and novel cancer therapies cause apoptosis both in vitro and in vivo.[50] Thus noninvasive markers of apoptosis may be useful in assessing and comparing efficacy of new therapeutic agents. During apoptosis, several cellular proteins are cleaved by caspases and then released from disintegrated apoptotic cells into the circulation. Cytokeratin 18 is an abundant

intracellular protein expressed by most carcinomas including prostate cancer.[51] During apoptosis of cytokeratin 18 expressing cells, cytokeratin 18 is cleaved by caspases resulting in exposure of a neo-epitope (CK18Asp396);[52] a validated marker of apoptosis.[51-53] Only apoptosis of cytokeratin 18 expressing cells will result in release of CK18Asp396 thus there is no cross-reactivity with apoptosis of lymphoid tissue, bone marrow, neuronal cells or other nonepithelial tissue. Levels of CK18Asp396 can be measured in serum using a commercially available ELISA, M30-Apoptosense® (PEVIVA AB). This ELISA utilizes the M30 monoclonal antibody (Cytodeath, Roche) which recognizes the neo-epitope that maps to positions 396 of the caspase cleaved fragment of cytokeratin 18. Use of this antibody in the format of immunohistochemistry to measure apoptosis has been shown to be superior to TUNEL and ISEL.[52, 54-58] More recently, the M30 antibody has been utilized as part of the M30-Apoptosense® ELISA allowing quantitative assessment of apoptosis of tumor cells from peripheral blood.[59, 60] Kramer et al recently utilized measurement of serum CK18Asp396 to assess efficacy of different anti-cancer drugs in treatment of prostate cancer.[61] Greater increases in CK18Asp396 were measured after treatment with docetaxel in comparison to treatment with vinorelbine and estramustine correlating with known efficacy of these agents. Thus the ability of serum levels of CK18Asp396 to provide a noninvasive measure of anti-tumor effect in prostate cancer warrants further investigation. In addition to assessment of anti-tumor effect, peripheral markers of apoptosis could also provide early evidence of treatment failure.

2.6 Study Rationale

Metastatic hormone refractory prostate cancer is a protracted morbid disease. As a major solid tumor affecting American men and contributing to a significant number of deaths, better therapeutics are a must. Therefore, better imaging technology leading to the ability to assess bone lesions in metastatic prostate cancer will no doubt lead to a significant advance in the field. As presented above, functional diffusion maps (fDM) have proven to be a sensitive measure of treatment response and an early biomarker of overall clinical outcomes in other tumor types. Preclinically we have successfully utilized this technology to assess treatment response of bone metastases using a PC3 prostate cancer xenograft model. This study will evaluate the **feasibility** of identifying changes in diffusion MRI characteristics of bone that correlate with response to therapy in men with metastatic prostate cancer.
The target population of this trial will be men with hormone refractory prostate cancer initiating treatment with chemotherapy.[2, 3] Only 30-50% of these patients are expected to respond by PSA measures.[2, 3] Therefore, in order to maximize treatment responses seen in this trial, hence our ability to assess the characteristics of response to therapy, we will also include a second cohort of men with newly diagnosed metastatic disease initiating therapy with androgen deprivation; a group with an expected 90% response rate to therapy.

To provide additional measures of the ability of functional diffusion maps to evaluate treatment response of bone metastases, we will also investigate markers of bone turnover and apoptosis. We hypothesize that markers of apoptosis will increase and bone turnover markers will decrease in response to therapy and that changes will correlate with clinical response and changes detected with functional diffusion mapping.

3. PATIENT SELECTION

3.1 Inclusion Criteria

All patients must have:

3.1.1 Diagnosis of prostate cancer.

3.1.2 Evidence of metastatic disease by bone scan.

** If the bone scan was done at an outside facility, a copy of the study must be submitted to the study radiologist.*

3.1.3 Patients must be initiating systemic therapy for metastatic disease.

a.) New D2 disease beginning therapy with androgen deprivation.

b.) CRPC beginning systemic non hormonal therapy.

3.1.4 Patients must be willing to provide the blood samples for the correlative markers.

3.1.5 Patients must be able to lie flat in an MRI magnet for 30-60 minutes.

3.1.6 Life expectancy of 12 weeks or greater.

3.1.7 All patients must be informed of the investigational nature of this study and must sign an informed consent in accordance with institutional and federal guidelines.

3.2 Exclusion Criteria

3.2.1 Contraindication to MRI imaging*.

3.2.2 Patients who require sedation with general anesthesia to undergo MRI imaging.

3.2.3 Weight greater than 275 pounds.

* See Appendix B for MRI guidelines for patients with surgical hardware/implants.

3.3 Inclusion of Women and Minorities

- This study applies only to men. Men of all races and ethnic groups are eligible for this trial.

4. REGISTRATION:

Patients satisfying the inclusion/exclusion criteria will be recruited into the study. After informed consent is obtained and prior to the initiation of protocol intervention, all patients must be first registered with the UM-CCC Clinical Trials Office. Please contact Amie Anderson, the data manager for this trial at (734) 615-1749, Monday-Friday, 8:15AM – 5:00PM ET. A faxed copy of the signed, institutionally approved informed consent will be needed to complete the registration process. UMCCC-CTO Fax number: (734) 232-0744. At the time of registration with the UMCCC-CTO the following information will be taken:

1. **Registering Individual** **[Last, First Name]**
2. **Consenting Physician** **[Last, First Name]**
3. **Participants Initials** **[First, Last Initial]**
4. **Participants DOB**
5. **Date of Signed Informed Consent**
6. **Status of Disease (New D2 or Androgen Independent)**
7. **Type and Timing of Systemic Therapy to be Initiated**

Once registration is complete, the Registrar will provide the registering individual a study subject identification number. This number must be included on all data submission forms.

5. PROTOCOL METHODS

5.1 Imaging Protocol:

MRI imaging will be scheduled by calling 734-936-4500 and requesting the study to be done on the 3T research-dedicated magnet. The MRI checklist (see appendix A.) must be completed before scheduling the MRI imaging. The patient’s height, weight, medical record number, study ID number and the protocol number will be needed at time of scheduling.

5.1.1 Diffusion MRI

- **Timing:** Patients will undergo diffusion MRI imaging at baseline, week two of therapy, and between weeks 9 and 11 of therapy.

- **Localization of region of interest:** In conjunction with bone scan images, limited standard MRI sequences including T1-weighted, proton density-weighted and STIR sequences of the pelvis and/or spine will be used to identify areas of interest by the study radiologist for diffusion MRI imaging. The clinical radiologist will be blinded to the clinical response and treatment.

- **Diffusion Magnetic Resonance Imaging Procedure:** All bone MRI examinations conducted on behalf of this project will be performed on the research-dedicated 3T Philips Achieva MRI system located within the UMHS MRI facility. The current imaging protocol is designed to allow use of

commercial multichannel phased array coils selected based on lesion anatomical location. Possible choices include Dual Flex M, Torso, and Cardiac coils which are all "SENSE-compatible", thus allow use of parallel imaging technology to reduce acquisition time and spatial distortion in single-shot EPI-based diffusion-weighted imaging (DWI) sequences.

The MRI protocol includes: 3-plane anatomical survey; SENSE reference; Proton-Density; STIR Fat-Suppressed; T1-weighted, and Diffusion-Weighted imaging (DWI). All but the DWI are routinely performed in clinical orthopedic examinations. Depending on lesion location, the applied MRI examination will be drawn from the closest related clinical MRI protocol with the addition of diffusion. Nominal parameter settings of the DWI sequence are: Axial DWI: FOV=350mm; AcqMatrix = 196x86; SENSE factor 2; 30slices; 4mm thick; 2D SE-EPI 3-axis DWI at b=0 and 1000sec/mm2; STIR (fat suppression) TR/TE/TI = 12000 / 62 / 150; NSA = 2; AcqTime = 4:41

Additional diffusion measurements may be acquired using diffusion MRI outside of the pelvis/lumber spine area in patients found to have significant disseminated tumor spread. These images would be used to determine the feasibility of a future study of whole body diffusion MRI for complete assessment of bone metastases. Additional images will be voluntary and clearly stated in the consent form.

- **Creation of functional diffusion maps (fDM):** fDM will be developed on all 20 patients at the week two exam and also at the later exam (9-11 weeks after initiation of therapy). Analysis of the fDM will be preformed by the study radiology physicist who will be blinded to clinical response and treatment.

Image / Data Processing Procedures

Full-exam image sets will be de-identified and transferred to an image processing workstation. A series of "in house" MatLab® scripts will be implemented to convert each series into a "vector" format suitable for automatic image registration. Image registration will be performed using an automated linear affine coregistration algorithm (MIAMI Fuse; University of Michigan, Ann Arbor, MI).[63] The vectorized format refers to each pixel within a volumetric scan having multiple values. Our current procedure is to assign the image with the highest average information content to the first vector value, with lower information data assigned to subsequent vector values. Once the optimal spatial transformation is determined, the transformation is mathematically applied to all vector values. In this way, even maps that contain nearly no spatial detail were spatially registered to the reference geometry. Each patient provides their own anatomic reference to which all their images will be spatially registered. The patients' T2-weighted images acquired before therapy serve as their anatomic reference.

Diffusion MRI Processing:

Each diffusion-weighted image series will be comprised of four images per anatomic slice: a "low b-value" image with relatively high SNR and T2-weighting, and three "high b-value" with high diffusion weighting in three orthogonal directions. The product of these three images exhibits strong sensitivity to diffusion but without sensitivity to the structural directionality of the tissues. This isotropic feature is crucial to follow serial changes in water diffusion without confounding effects due to tissue orientation. The logarithm of the ratio of low-b (S_{b0}) and high-b (S_{b1000}) images, performed on a pixel-by-pixel basis, and scaled by the known b-value difference will be used to yield an ADC map for each anatomic section as follows:

$$ADC = \frac{mm^2}{1000\,\mathrm{sec}} \log\left[\frac{S_{b0}}{S_{b1000}}\right]$$

ADC maps will be generated offline via our own Matlab® routines or I-Response software (Cedara Software Inc.) which store results as a three-dimensional (i.e. x, y, z image), three-vector maps (low b-value, high b-value, ADC).

ADC histogram and fDM analyses:

Using a region of interest (ROI) drawing tool, the tumor margins will be defined on the basis of abnormalities seen on the T2, T1 and T1 enhanced images by the study radiologist. From these ROIs the cross sectional diameter product (CDP) will be calculated, and the ADC histogram and fDM analyses will be performed. Graphical superposition of pre-and post-treatment ADC histograms will provide a visual sense of change in the distribution of ADC values, but for statistical analysis only, the mean of the ADC histogram will be used. The fDM analysis will be used to produce the fDM maps and scatter plots as previously described. [27] Briefly, the post treatment ADC value of each voxel within both ROIs will be plotted as a function of its pre-treatment ADC value. Voxels where the ADC value in the follow up scan has increased (red voxels) and decreased (blue voxels) significantly from baseline will be segmented from the rest of the tumor (green voxels) to calculate the two fDM volumes: V_R (sum of red voxels) and V_B (sum of blue voxels), respectively. The threshold of a significant increase or decrease in pixel ADC value was $0.55 \times 10^{-3} mm^2/sec$ based upon previous studies. [27, 28, 64]

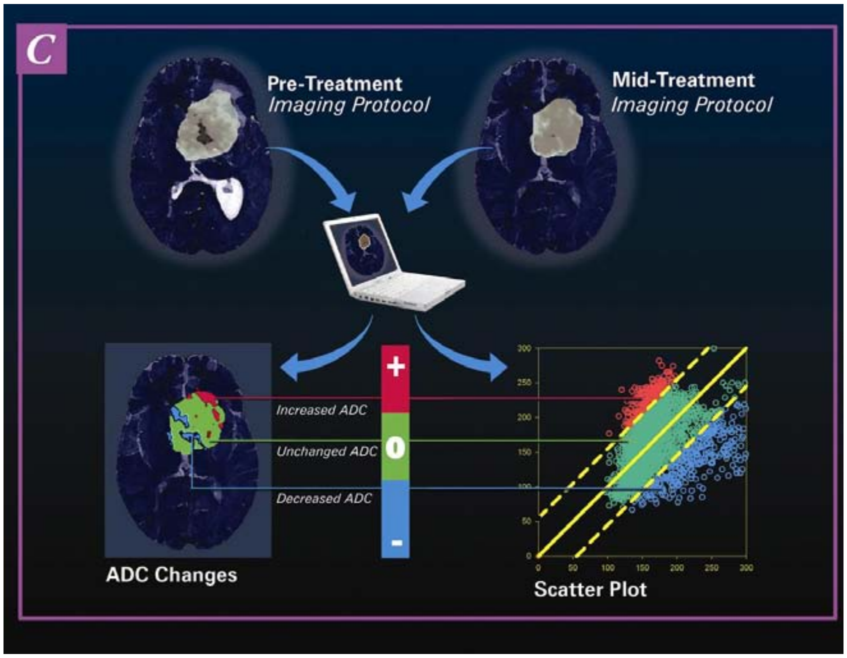

Representative schematic of functional diffusion mapping. Diffusion MRI data undergoes digital image post-processing and analysis which involves co-registration of images before and during treatment. Data is used to generate a 3-color overlay representing regions in which tumor ADC values are unchanged (green voxels), significantly increased (red voxels) or significantly decreased (blue voxels). This data can also be presented in a scatter plot and percentages assigned to 3 defined ADC regions allowing quantitative assessment of overall changes in tumor ADC values.

There is potential that clinically relevant data will be obtained from the imaging used to determine the location of interest. The treating physicians will have access to the results of the anatomic imaging (not the fDM) and will have the opportunity to base clinical decisions on this imaging. The potential for clinically relevant data to be obtained from the baseline imaging will be discussed with the subjects and included in the informed consent.

5.2 Correlative Studies Procedures and Methods:

5.2.1 Blood draws will be performed on day 1, during week 2 of therapy, and between weeks 9 and 12 for correlative studies.

5.2.2 Blood: Approximately 10 ml of blood will be drawn into 2 standard serum separator tubes at each time point. The blood will be spun at 3000 RPM for 10 minutes in a clinical centrifuge. The serum (top layer above clot) will be harvested immediately using a pipettor and saved in equal aliquots in self-standing microcentrifuge tubes (Fisher Scientific) labeled with the patient's study I.D. number and date of sample collection. Microcentrifuge tubes will be taken to the Translational Research Lab where they will be frozen and stored at -80 °C until used. **(please use submission form provided in appendix C)**

Contact in Translational Research Lab
Cliff Abat
Room 2534 Cancer Center
1500 East Medical Center Dr
Ann Arbor, MI 48109-5946

Email: abatcn@med.umich.edu
Phone (734) 615-5726

The following correlative studies will be performed using blood samples.

- **CK18Asp396:** During apoptosis cytokeratin 18 is cleaved resulting in exposure of a neo-epitope (CK18Asp396) which is a validated marker of apoptosis.[51-53] Serum CK18Asp396 will be measured using a commercially available ELISA, M30-Apoptosense® (PEVIVA AB, Bromma, Sweden) per manufacturer's instructions. Inter-assay variability is 2.9% and intra-assay variability is 4.9%. The assay range is 75-1,000 Units/L. The detection limit of the assay is 30 Units/L.

- **Bone-specific alkaline phosphatase (BAP):** BAP is a protein produced by osteoblasts that is an indicator of bone production. Bone-specific BAP will be measured using a commercially available ELISA, Metra® BAP (Metra Biosystems: Quidel Corporation, Mountain View, CA) per manufacturer's instructions. Intra-assay variability is 4-6% and inter-assay variability is 5-8%. The assay range is 2-140 units/L. The detection limit of the assay is 0.7 units/L.

- **Serum N-telopeptide (NTx):** Cross-linked N-telopeptides of bone type I collagen are released into the serum as a result of osteoclast activity on bone thus providing a specific biochemical indicator of bone resorption. Serum Ntx will be measured using a commercially available ELISA, Osteomark NTX® (Wampole Laboratories, Princeton, NJ) per manufacturer's instructions. Intra-assay variability is 4.6% and inter-assay variability is 6.9%. Antigen recovery ranges from 94-105%. The assay range is 3.2-40 mM Bone Collagen Equivalents (BCE). The detection limit of the assay is 3.2 BCE.

6. DURATION OF STUDY

- Patients will be followed for 1 year or until disease progression; which ever is achieved first. Follow-up will be in the form of medical record review.

7. CRITERIA FOR REMOVAL FROM STUDY:

7.1 Patients will be removed from study based on any of the following criteria:

- Patients wishes
- Cessation of systemic therapy
- Change in treatment regimen
- Intercurrent illness that prevents further administration of treatment
- General or specific changes in the patient's condition that render the patient unacceptable for further treatment in the judgment of the investigator
- Disease progression

8. STATISTICAL CONSIDERATIONS

8.1 Study Design/Endpoints

As this is a pilot study, no formal sample size/power calculations will be performed. Further, no formal statistical hypotheses will be tested. We will accrue 15 HRPCa patients and 5 hormone therapy naïve patients for this study. Functional diffusion maps (fDM) will be developed on all 20 patients at the week two exam and also at the later exam (9-11 weeks after initiation of therapy). The fDM methods developed by Moffat, et.al[11] will be used in this study. fDM from metastatic lesions in CRPCa patients will be of primary interest. However, given the small sample size inherent to a pilot study and only a 30-50% expected response rate to therapy in patients with CRPCa, fDM from metastatic lesions in hormone therapy naïve patients will be included. Given an expected overall response rate of ~90% in this patient population, inclusion of this cohort of patients will maximize treatment responses seen in this pilot trial and thus our ability to characterize fDM characteristics of prostate cancer bone metastases and changes seen in response to therapy.

We will compute summary statistics of the data including means, medians, range, quartiles and standard deviations. Scatterplots and histograms will also be plotted. Several endpoints of interest will be used to investigate the utility of fDM as an early predictor to therapy in prostate cancer. First, clinically assessed time-to-progression (TTP) will be an outcome of interest. We will fit Cox proportional hazards model to the TTP with fDM as a covariate. Other covariates may include patient age and baseline PSA levels.

Secondly, we will also consider the correlation of fDM with blood correlates at both weeks two and between weeks 9 and 12. For these correlative studies we will use Pearson's correlation or Spearman's rank correlation to determine if fDM correlates with with best response status and serum biomarkers.

The fDM method outlined in Moffat et.al. uses the 95% confidence intervals of healthy tissue ADC values during therapy regressed on those pre-therapy as thresholds to define tumor regions that have significant changes in ADC. This may not be the most optimal threshold for defining the fDM map. Therefore, we will perform ROC analysis by changing the thresholds used to define the fDM map. Truth will be dichotomized by clinical outcome for ROC purposes. The two outcomes will be progressors versus the combination of stable disease and responders.

Upon establishing that changes in fDM of bone metastases occur with therapy, results from this study will be used to design and power a larger more definitive study.

For the correlative studies we will use Pearson's correlation or Spearman's rank correlation to determine if fDM correlates with biomarkers of apoptosis and with biomarkers of bone turnover.

We anticipate that there may be several lesion sites per subject that are analyzed. Initially, we will treat all lesions as independent observations. We will then consider a random effects ANOVA model to determine if there is a mean difference of fDM between the three groups defined by a drop in PSA. We will include a single subject specific random effect in the model, with lesions nested within subjects. Furthermore, since PSA and the biomarkers of apoptosis and bone turnover are measured at the individual level, and fDM measures response within individual lesions, we will consider the average fDM measure of all lesions within a subject and correlate that with PSA and with biomarkers of apoptosis and bone turnover.

8.2 Sample Size/Accrual Rate

- This trial will accrue 20 patients. We anticipate accrual to take 18 months.

9. STUDY CALENDAR:

A. Study Specific

	**Baseline**	**Week 2**	**Between weeks 9 and 12**
Diffusion MRI Imaging	X	X	X
Blood for Correlative Studies	X	X	X

B. Standard of Care

Data for these assessments will be taken from the patient medical records. These assessments are part of routine care and will not be done for the purpose of this study. This information will be used to determine treatment response.

	**Baseline**	**Around weeks 9- 12**
History and Physical Exam	X	X
Appropriate imaging to assess all sites of disease as clinically indicated	X	X
Bone Scan	X	X
PSA	X	X
Overall Best Response Status**		X

**Complete response, partial response, stable disease, or progressive disease as measured by classical clinical endpoints (see section 12)

10. MEASUREMENT OF TREATMENT EFFECT

Patients' charts will be used to obtain data outlined in the Standard of Care (B) Study Calendar Table above to determine response to treatment to be correlated with protocol imaging studies.

PSA declines will be the primary parameter use to define "response" in this study. However, PSA response will be coded as response only in the presence of at least stable disease by all other objective disease measures (see below). The consensus guidelines[4] recommended that outcome be categorized for disease measures. Therefore, to provide consistency, in this trial we are including definitions of outcome for all disease measures.

10.1 Outcomes based on post-therapy PSA changes:

10.1.1 Complete Response (CR): Undetectable PSA (<0.2 ng/ml) that is confirmed by another PSA level at no less than 4 weeks.

10.1.2 Partial Response (PR): Decrease in PSA value by $\geq$ 50% that is confirmed by another PSA level at no less than 4 weeks.

10.1.3 Stabilization (SD): Patients who do not meet the criteria for CR, PR or PD will be considered stable

10.1.4 Progression (PD): 25% increase over baseline or nadir whichever is lower and an increase in the absolute value of PSA level by 5 ng/ml that is confirmed by another PSA level at no less than 4 weeks.

10.2 Outcomes based on Measurable disease:

Response and progression criteria will be based on RECIST definitions.[65]

10.3 Outcomes based on Radionuclide bone scans:

The subjectivity in interpreting serial changes in a radionuclide bone scan is well recognized. Thus, the primary outcome will be whether the scan is stable or improved, vs. worse or progression. Changes in intensity will not be used as an outcome measure.

10.3.1 Stable or Improved: A stable or improved classification requires that no new lesions appear or that new pain has not developed in an area that was previously visualized.

10.3.2 Progression (Non-Response): Appearance of **two or more** new skeletal lesions. *An increase in the size or intensity of known skeletal lesions will not be considered progression*.

10.4 Definition of Progression Based on Pain:

10.4.1 Progression by pain criteria is based on pain due to prostate cancer requiring one or more of the following palliative interventions:

Opioid Therapy: Intravenous, intramuscular or subcutaneous opioid therapy administered as a single dose; oral or transdermal opioid analgesic use administered for 10 out of 14 consecutive days, and/or requiring **Radionuclide or Radiation therapy.**

10.4.2 Evidence of disease at the site of pain is required. Pain requiring only non-opioid analgesics will not be considered disease progression.

11. REGULATORY AND REPORTING REQUIREMENTS

- Only adverse events directly related to the protocol experimental methods will be reported. The descriptions and grading scales found in the revised NCI Common Terminology Criteria for Adverse Events (CTCAE) version 3.0 will be utilized for adverse event reporting.

- SAE Definition. A SAE is any adverse event, without regard to causality, that is life-threatening or that results in any of the following outcomes: death; in-patient hospitalization or prolongation of existing hospitalization; persistent or significant disability or incapacity; or a congenital anomaly or birth defect. Any other medical event that, in the medical judgment of the Principal Investigator, may jeopardize the subject or may require medical or surgical intervention to prevent one of the outcomes listed above is also considered an SAE. A planned medical or surgical procedure is not, in itself, an SAE. Also specifically excluded from this definition of SAE is any event judged by the Principal Investigator to represent progression of the malignancy under study, unless it results in death within the SAE Reporting Period.

- SAE Reporting Period. The SAEs that are subject to this reporting provision are those that occur from the time of registration through 30 days after completion of protocol procedures.

- SAE reporting will be done through eResearch. Instructions to create an adverse event within eResearch can be found at http://www.umich.edu/~eresinfo/docs/ae.pdf.

  Note: All deaths on study must be reported using expedited reporting regardless of causality. Attribution to treatment or other cause should be provided.

11.1 Data Reporting

- All data must be entered in “real time” such that all data are current.

12. DATA MANAGEMENT AND MONITORING:

12.1 DATA COLLECTION/ENTRY:

The data collected for this study will be entered into the Velos eResearch Database which is secure. Source documentation will be available to support the computerized patient record.

Adverse events, including all toxic effects of treatment, will be tabulated, individually summarized by body system and to severity or toxicity grade. <u>All expedited AE reports will be submitted to the University of Michigan IRB.</u>

12.2 DATA SUBMISSION: The data collected for this study will be entered into the Velos e-Research database which is a secure database

12.3 DATA SECURITY AND CONFIDENTIALITY: To ensure security and confidentiality, participant names are not part of the research record. Participant initials, date of birth and a study-specific identification number are the sole identifiers. Physical security and systematic backup for the centralized database is provided by a professionally managed and equipped data center with tightly controlled access. Access to data rigorously complies with HIPAA requirements, governed by HIPAA's "minimum necessary" principle. The software provides audit trails on all data entry and modification of existing data. Source documentation will be available to support the computerized patient record.

12.4 QUALITY ASSURANCE/DATA AND SAFETY MONITORING: Monthly registration reports will be generated to monitor patient accruals and completeness of registration data. Data queries will be generated for the data managers and investigators when there are questions or incomplete data. Routine data quality reports will be generated to assess missing data and inconsistencies. Accrual rates and extent and accuracy of evaluations and follow-up will be monitored periodically throughout the study period and potential problems will be brought to the attention of the study team for discussion and action.

12.4.1 Data Safety and Monitoring: There will be scheduled meetings that will be conducted monthly. These meetings will include the protocol investigators and data managers involved with the conduct of the protocol.

During these meetings the investigators will discuss matters related to:

1. Safety of protocol participants (AE reporting).
2. Validity and integrity of the data.
3. Enrollment rate relative to expectation, characteristics of participants
4. Retention of participants, adherence to protocol (potential or real protocol violations).
5. Data completeness.

12.4.2 Audits and Data Completeness

There will be random-sample data quality and protocol compliance audits for patients registered for this specific trial conducted by UMCCC Quality Assurance Review Committee (QARC). These audits will occur at a minimum of one time per year, more frequently if indicated. All data on all enrolled study patients within the University of Michigan Velos eResearch Database will be examined for protocol compliance.

13. REFERENCES

1. Jemal A, Siegel R, Ward E, et al. Cancer statistics, 2006. CA Cancer J Clin 2006;56(2):106-30.

2. Petrylak DP, Tangen CM, Hussain MH, et al. Docetaxel and estramustine compared with mitoxantrone and prednisone for advanced refractory prostate cancer. N Engl J Med 2004;351(15):1513-20.

3. Tannock IF, de Wit R, Berry WR, et al. Docetaxel plus prednisone or mitoxantrone plus prednisone for advanced prostate cancer. N Engl J Med 2004;351(15):1502-12.

4. Bubley GJ, Carducci M, Dahut W, et al. Eligibility and response guidelines for phase II clinical trials in androgen-independent prostate cancer: recommendations from the Prostate-Specific Antigen Working Group. J Clin Oncol 1999;17(11):3461-7.

5. Carducci M, Nelson JB, Saad F, et al. Effects of atrasentan on disease progression and biological markers in men with metastatic hormone-refractory prostate cancer: Phase 3 study. J Clin Oncol 2004;22(14S):4508.

6. Vogelzang N, Nelson J, Schulman C, et al. Meta-analysis of clinical trials of atrasentan 10 mg in metastatic hormone-refractory prostate cancer. J Clin Oncol 2005;23(16S):4563.

7. Pollen JJ, Witztum KF, Ashburn WL. The flare phenomenon on radionuclide bone scan in metastatic prostate cancer. AJR Am J Roentgenol 1984;142(4):773-6.

8. Stokkel MP, Valdes Olmos RA, Hoefnagel CA, Richel DJ. Tumor and therapy associated abnormal changes on bone scintigraphy. Old and new phenomena. Clin Nucl Med 1993;18(10):821-8.

9. Vogel CL, Schoenfelder J, Shemano I, Hayes DF, Gams RA. Worsening bone scan in the evaluation of antitumor response during hormonal therapy of breast cancer. J Clin Oncol 1995;13(5):1123-8.

10. Janicek MJ, Hayes DF, Kaplan WD. Healing flare in skeletal metastases from breast cancer. Radiology 1994;192(1):201-4.

11. Johns WD, Garnick MB, Kaplan WD. Leuprolide therapy for prostate cancer. An association with scintigraphic "flare" on bone scan. Clin Nucl Med 1990;15(7):485-7.

12. Levenson RM, Sauerbrunn BJ, Bates HR, Newman RD, Eddy JL, Ihde DC. Comparative value of bone scintigraphy and radiography in monitoring tumor response in systemically treated prostatic carcinoma. Radiology 1983;146(2):513-8.

13. Coleman RE, Rubens RD. The clinical course of bone metastases from breast cancer. Br J Cancer 1987;55(1):61-6.

14. Ross BD, Chenevert TL, Kim B, al. E. Magnetic Resonance Imaging and Spectroscopy: Application to Experimental Neuro-Oncology. Q Magn Reson Biol Med 1994;1:89-106.

15. Chenevert TL, McKeever PE, Ross BD. Monitoring early response of experimental brain tumors to therapy using diffusion magnetic resonance imaging. Clin Cancer Res 1997;3(9):1457-66.

16. Zhao M, Pipe JG, Bonnett J, Evelhoch JL. Early detection of treatment response by diffusion-weighted 1H-NMR spectroscopy in a murine tumour in vivo. Br J Cancer 1996;73(1):61-4.

17. Lemaire L, Howe FA, Rodrigues LM, Griffiths JR. Assessment of induced rat mammary tumour response to chemotherapy using the apparent diffusion coefficient of

tissue water as determined by diffusion-weighted 1H-NMR spectroscopy in vivo. Magma 1999;8(1):20-6.

18. Poptani H, Puumalainen AM, Grohn OH, et al. Monitoring thymidine kinase and ganciclovir-induced changes in rat malignant glioma in vivo by nuclear magnetic resonance imaging. Cancer Gene Ther 1998;5(2):101-9.

19. Hakumaki JM, Poptani H, Puumalainen AM, et al. Quantitative 1H nuclear magnetic resonance diffusion spectroscopy of BT4C rat glioma during thymidine kinase-mediated gene therapy in vivo: identification of apoptotic response. Cancer Res 1998;58(17):3791-9.

20. Geschwind JF, Artemov D, Abraham S, et al. Chemoembolization of liver tumor in a rabbit model: assessment of tumor cell death with diffusion-weighted MR imaging and histologic analysis. J Vasc Interv Radiol 2000;11(10):1245-55.

21. Stegman LD, Rehemtulla A, Hamstra DA, et al. Diffusion MRI detects early events in the response of a glioma model to the yeast cytosine deaminase gene therapy strategy. Gene Ther 2000;7(12):1005-10.

22. Galons JP, Altbach MI, Paine-Murrieta GD, Taylor CW, Gillies RJ. Early increases in breast tumor xenograft water mobility in response to paclitaxel therapy detected by non-invasive diffusion magnetic resonance imaging. Neoplasia 1999;1(2):113-7.

23. Jennings D, Hatton BN, Guo J, et al. Early response of prostate carcinoma xenografts to docetaxel chemotherapy monitored with diffusion MRI. Neoplasia 2002;4(3):255-62.

24. Brunberg JA, Chenevert TL, McKeever PE, et al. In vivo MR determination of water diffusion coefficients and diffusion anisotropy: correlation with structural alteration in gliomas of the cerebral hemispheres. AJNR Am J Neuroradiol 1995;16(2):361-71.

25. Moffat BA, Hall DE, Stojanovska J, et al. Diffusion imaging for evaluation of tumor therapies in preclinical animal models. Magma 2004;17(3-6):249-59.

26. Chenevert TL, Stegman LD, Taylor JM, et al. Diffusion magnetic resonance imaging: an early surrogate marker of therapeutic efficacy in brain tumors. J Natl Cancer Inst 2000;92(24):2029-36.

27. Moffat BA, Chenevert TL, Lawrence TS, et al. Functional diffusion map: a noninvasive MRI biomarker for early stratification of clinical brain tumor response. Proc Natl Acad Sci U S A 2005;102(15):5524-9.

28. Hamstra DA, Chenevert TL, Moffat BA, et al. Evaluation of the functional diffusion map as an early biomarker of time-to-progression and overall survival in high-grade glioma. Proc Natl Acad Sci U S A 2005;102(46):16759-64.

29. Zhou XJ, Leeds NE, McKinnon GC, Kumar AJ. Characterization of benign and metastatic vertebral compression fractures with quantitative diffusion MR imaging. AJNR Am J Neuroradiol 2002;23(1):165-70.

30. Park SW, Lee JH, Ehara S, et al. Single shot fast spin echo diffusion-weighted MR imaging of the spine; Is it useful in differentiating malignant metastatic tumor infiltration from benign fracture edema? Clin Imaging 2004;28(2):102-8.

31. Pui MH, Mitha A, Rae WI, Corr P. Diffusion-weighted magnetic resonance imaging of spinal infection and malignancy. J Neuroimaging 2005;15(2):164-70.

32. Baur A, Stabler A, Bruning R, et al. Diffusion-weighted MR imaging of bone marrow: differentiation of benign versus pathologic compression fractures. Radiology 1998;207(2):349-56.

33. Saad F, Gleason DM, Murray R, et al. A randomized, placebo-controlled trial of zoledronic acid in patients with hormone-refractory metastatic prostate carcinoma. J Natl Cancer Inst 2002;94(19):1458-68.
34. Brown JE, Cook RJ, Major P, et al. Bone turnover markers as predictors of skeletal complications in prostate cancer, lung cancer, and other solid tumors. J Natl Cancer Inst 2005;97(1):59-69.
35. Coleman RE, Major P, Lipton A, et al. Predictive value of bone resorption and formation markers in cancer patients with bone metastases receiving the bisphosphonate zoledronic acid. J Clin Oncol 2005;23(22):4925-35.
36. Keller ET, Brown J. Prostate cancer bone metastases promote both osteolytic and osteoblastic activity. J Cell Biochem 2004;91(4):718-29.
37. Berruti A, Piovesan A, Torta M, et al. Biochemical evaluation of bone turnover in cancer patients with bone metastases: relationship with radiograph appearances and disease extension. Br J Cancer 1996;73(12):1581-7.
38. Vinholes J, Coleman R, Eastell R. Effects of bone metastases on bone metabolism: implications for diagnosis, imaging and assessment of response to cancer treatment. Cancer Treat Rev 1996;22(4):289-331.
39. Percival RC, Urwin GH, Harris S, et al. Biochemical and histological evidence that carcinoma of the prostate is associated with increased bone resorption. Eur J Surg Oncol 1987;13(1):41-9.
40. Zhang J, Dai J, Qi Y, et al. Osteoprotegerin inhibits prostate cancer-induced osteoclastogenesis and prevents prostate tumor growth in the bone. J Clin Invest 2001;107(10):1235-44.
41. Carlin BI, Andriole GL. The natural history, skeletal complications, and management of bone metastases in patients with prostate carcinoma. Cancer 2000;88(12 Suppl):2989-94.
42. Karsenty G. Bone formation and factors affecting this process. Matrix Biol 2000;19(2):85-9.
43. Parfitt AM. The mechanism of coupling: a role for the vasculature. Bone 2000;26(4):319-23.
44. Boyce BF, Hughes DE, Wright KR, Xing L, Dai A. Recent advances in bone biology provide insight into the pathogenesis of bone diseases. Lab Invest 1999;79(2):83-94.
45. Pectasides D, Nikolaou M, Farmakis D, et al. Clinical value of bone remodelling markers in patients with bone metastases treated with zoledronic acid. Anticancer Res 2005;25(2B):1457-63.
46. Carducci MA, Padley RJ, Breul J, et al. Effect of endothelin-A receptor blockade with atrasentan on tumor progression in men with hormone-refractory prostate cancer: a randomized, phase II, placebo-controlled trial. J Clin Oncol 2003;21(4):679-89.
47. Lara PN, Jr., Stadler WM, Longmate J, et al. A randomized phase II trial of the matrix metalloproteinase inhibitor BMS-275291 in hormone-refractory prostate cancer patients with bone metastases. Clin Cancer Res 2006;12(5):1556-63.
48. Kerr JF, Wyllie AH, Currie AR. Apoptosis: a basic biological phenomenon with wide-ranging implications in tissue kinetics. Br J Cancer 1972;26(4):239-57.
49. Bortner CD, Cidlowski JA. Apoptotic volume decrease and the incredible shrinking cell. Cell Death Differ 2002;9(12):1307-10.

50. Gerl R, Vaux DL. Apoptosis in the development and treatment of cancer. Carcinogenesis 2005;26(2):263-70.
51. Kramer G, Erdal H, Mertens HJ, et al. Differentiation between cell death modes using measurements of different soluble forms of extracellular cytokeratin 18. Cancer Res 2004;64(5):1751-6.
52. Leers MP, Kolgen W, Bjorklund V, et al. Immunocytochemical detection and mapping of a cytokeratin 18 neo-epitope exposed during early apoptosis. J Pathol 1999;187(5):567-72.
53. Cummings J, Ward TH, LaCasse E, et al. Validation of pharmacodynamic assays to evaluate the clinical efficacy of an antisense compound (AEG 35156) targeted to the X-linked inhibitor of apoptosis protein XIAP. Br J Cancer 2005;92(3):532-8.
54. Carr NJ. M30 expression demonstrates apoptotic cells, correlates with in situ end-labeling, and is associated with Ki-67 expression in large intestinal neoplasms. Arch Pathol Lab Med 2000;124(12):1768-72.
55. Kusama K, Jiang Y, Ohno J, et al. Immunohistochemical detection of cytokeratin 18 and its neo-epitope in human salivary glands and pleomorphic adenomas. Anticancer Res 2000;20(4):2485-7.
56. Rupa JD, de Bruine AP, Gerbers AJ, et al. Simultaneous detection of apoptosis and proliferation in colorectal carcinoma by multiparameter flow cytometry allows separation of high and low-turnover tumors with distinct clinical outcome. Cancer 2003;97(10):2404-11.
57. Kadyrov M, Kaufmann P, Huppertz B. Expression of a cytokeratin 18 neo-epitope is a specific marker for trophoblast apoptosis in human placenta. Placenta 2001;22(1):44-8.
58. Mirzaie-Joniani H, Eriksson D, Sheikholvaezin A, et al. Apoptosis induced by low-dose and low-dose-rate radiation. Cancer 2002;94(4 Suppl):1210-4.
59. Biven K, Erdal H, Hagg M, et al. A novel assay for discovery and characterization of pro-apoptotic drugs and for monitoring apoptosis in patient sera. Apoptosis 2003;8(3):263-8.
60. Ueno T, Toi M, Biven K, Bando H, Ogawa T, Linder S. Measurement of an apoptotic product in the sera of breast cancer patients. Eur J Cancer 2003;39(6):769-74.
61. Kramer G, Schwarz S, Hagg M, Havelka AM, Linder S. Docetaxel induces apoptosis in hormone refractory prostate carcinomas during multiple treatment cycles. Br J Cancer 2006;94(11):1592-8.
62. Takahara T, Imai Y, Yamashita T, Yasuda S, Nasu S, Van Cauteren M. Diffusion weighted whole body imaging with background body signal suppression (DWIBS): technical improvement using free breathing, STIR and high resolution 3D display. Radiat Med 2004;22(4):275-82.
63. Meyer CR, Boes JL, Kim B, et al. Demonstration of accuracy and clinical versatility of mutual information for automatic multimodality image fusion using affine and thin-plate spline warped geometric deformations. Med Image Anal 1997;1(3):195-206.
64. Moffat BA, Chenevert TL, Meyer CR, et al. The functional diffusion map: an imaging biomarker for the early prediction of cancer treatment outcome. Neoplasia 2006;8(4):259-67.
65. Therasse P, Arbuck SG, Eisenhauer EA, et al. New guidelines to evaluate the response to treatment in solid tumors. European Organization for Research and Treatment

of Cancer, National Cancer Institute of the United States, National Cancer Institute of Canada. J Natl Cancer Inst 2000;92(3):205-16.

Appendix A. MRI Checklist

UNIVERSITY OF MICHIGAN HOSPITALS & HEALTH CENTERS<br>DEPARTMENT OF RADIOLOGY<br><br>PATIENT QUESTIONNAIRE FOR MR	BIRTHDATE<br><br>NAME<br><br>REG NO

Patient's Height: ______ ft ______ in. Weight: _______ kg **Travel:** ☐ Wheelchair ☐ Stretcher ☐ SWAT

Form completed by: ☐ Patient ☐ Family (relationship): ____________________ ☐ UMHHS Staff ☐ Other: ________________

DO YOU HAVE A HISTORY OF ANY OF THE FOLLOWING:	YES	NO	UNKNOWN
1. Any IV contrast (X-ray dye) allergy?	☐	☐	☐
2. A severe food or medication allergy?	☐	☐	☐
3. Are you pregnant or nursing (lactating)?	☐	☐	☐
4. Medical conditions: Sickle cell anemia	☐	☐	☐
5. Kidney failure/Renal Transplant	☐	☐	☐
6. Creatinine_________ Bun___________	☐	☐	☐
7. Cancer Type _____________	☐	☐	☐
8. Are you on Oxygen or a Ventilator	☐	☐	☐
9. Have you experience any problem(s) related to a previous MR Procedure	☐	☐	☐
10. Any surgery in the last 2 weeks or any previous surgery? If yes, what kind:____	☐	☐	☐
11.Have you had any of the following surgeries/implants/devices	☐	☐	☐
Cardiac pacemaker, pacemaker or pacemaker wires	☐	☐	☐
Any type of heart surgery, artificial heart valve	☐	☐	☐
Brain aneurysm surgery or other aneurysm clips	☐	☐	☐
Cochlear, otologic, middle ear or other ear implants	☐	☐	☐
Cataract surgery/eye lens implant	☐	☐	☐
Mechanical/electrical/stimulators/pumps or devices	☐	☐	☐
Neurostimulator/deep brain stimulator, vagal nerve stimulator	☐	☐	☐
Artificial limb or prosthesis	☐	☐	☐
Metal tracheostomy	☐	☐	☐
Have you ever been a machinist, welder or metal worker	☐	☐	☐
Have you ever had a piece of metal flushed/removed from your eye	☐	☐	☐
12. Can you stand without assistance	☐	☐	☐
13.Are you claustrophobic	☐	☐	☐
14.Are you physically/mentally impaired/unresponsive	☐	☐	☐
15.Do you have uncontrollable shaking or breathing problems	☐	☐	☐
16.Can you lie still on your back for 1 hour	☐	☐	☐

17.Do you have any of the following items inside of or on your body

☐ Bullets/BB's/pellets/shrapnel	☐ Shunts/stents/coils/filters	☐ **Dental implants**	☐ **Bone/neuro stimulator**
☐ Tattoo/permanent make-up	☐ Programmable shunts	☐ IUD/Prosthesis (penile, etc)	☐ **Vascular access port/catheter**
☐ Metal fragments	☐ **EKG patches**	☐ Breast implants	☐ **Foley with temperature probe**
☐ Surgical clips/staples	☐ **Transdermal med patches**	☐ Blood clot filter	☐ **Swan Ganz catheter**
☐ Wires/plates/screws/pins	☐ **Hearing aid**	☐ **Insulin/infusion/IV pump**	☐ **Dentures/ retainers/braces**

IMPORTANT INSTRUCTIONS: THE MR SYSTEM MAGNET IS ALWAYS ON!!!

PLEASE REMOVE ALL BODY PIERCING/JEWLERY/HAIRPINS AND OTHER METAL OBJECTS BEFORE ENTERING THE MR PROCEDURE ROOM. YOU WILL BE REQUIRED TO CHANGE INTO A GOWN AND WEAR EARPLUGS FOR SAFETY REASONS.

Signature of person completing this questionnaire Date

Signature of MR Staff reviewing form Date

Completed form:
Adults (18+): Tube to E6 OR
FAX to 734-647-7978
Peds (<18): Tube to N6 OR
FAX to 734-763-2421

Page 1 of 1

POD-0023	REV: 01/06<br>MIS: 01/06	White - Medical Record	University of Michigan Hospitals and Health Centers	PATIENT QUESTIONNAIRE FOR MR

Appendix B: Guidelines for MRI compatibility of Surgical Hardware/Implants.

Patients with the following items should not undergo MRI without the staff radiologist's approval.

1. Certain implanted electronic devices, pacemakers, internal defibrillators, cochlear implants, nerve and bone stimulators.
2. Cerebral aneurysm clips (Aneurysm clips put in at the U of M after 1985 are MRI compatible).
3. Cardiac or epicardial pacer wires – consultation with radiologist.
4. Penile implants - consultation with radiologist.
5. Swan Ganz catheters - must be removed.
6. Ferromagnetic foreign bodies in sensitive locations (such as brain, spine or orbital area) - direct consult with radiologist.
7. Metal tracheostomy - must be removed.
8. Ferromagnetic eyelid springs, magnetically activated tissue expanders –must be removed.
9. I.C.U. related appliances that are not MRI compatible must be removed.
10. Metallic stapedectomy prosthesis, tympanostomy tubes - consult with radiologist.
11. **Any metallic implant in place less than six weeks-consult with radiologist.**

It is generally safe to scan anyone with the following devices:

1. Internal orthopedic hardware (screws, plates, rods, nails, wires, and artificial joints).
2. Surgical clips, other than aneurysm clips, gut or skin staples, wires, mesh, sutures (time of surgery doesn't matter)
3. Diaphragms, IUDs, tubal ligation clips.
4. Non metallic penile prostheses.
5. Intravascular stents, coils, and IVC filters (if implanted longer than 6 weeks). (Green field stainless steel filters are OK to be placed in the magnet immediately after insertion.)
6. Intercranial pressure monitor.
7. Lens implants, scleral bands.
8. Neurosurgical CSF shunts (VP shunts, VA shunts, lumbar shunts, ventriculostomy catheters).
9. Synchromed infusion pumps.
10. Metal cardiac valves.

Appendix C

SPECIMEN COLLECTION FORM

Diffusion MRI Study

Patient I.D.:______________________Study Patient Initials: _________
Study Enrollment Date:_____________________________________
mm/dd/yyyy

Date of Sample Collection:____________________________
mm/dd/yyyy

Time of Collection:__Baseline___
Week 2___
Between weeks 9-12____

______Serum: Number of self-standing microcentrifuge tubes

UM Laboratory Contact:

Cliff Abat
Room 2534 Cancer Center
1500 East Medical Center Dr.
Ann Arbor, MI 48109-5946
Email abatcn@med.umich.edu
Phone (734) 615-5726

Date and Time Sample Received: ______________________________________
